# Psychophysiology of facial emotion recognition in psychopathy dimensions and oxytocin’s role: A scoping review

**DOI:** 10.1371/journal.pone.0327764

**Published:** 2025-07-30

**Authors:** Sara Ferreira-Nascimento, Filipa Freire, Diana Prata

**Affiliations:** 1 Instituto de Biofísica e Engenharia Biomédica, Faculdade de Ciências, Universidade de Lisboa, Portugal; 2 Egas Moniz School of Health and Science, Caparica, Portugal; 3 Institute of Psychiatry, Psychology and Neuroscience, King’s College London, United Kingdom; University of Pisa School of Engineering: Universita degli studi di Pisa Scuola di Ingegneria, ITALY

## Abstract

Psychopathy is characterized by social impairments that hinder effective societal functioning. It comprises two main dimensions: “Interpersonal-affective” and “Lifestyle-antisocial,” each associated with distinct patterns of traits and central and peripheral neurocorrelates, particularly concerning social salience and oxytocin function. In this review, we systematically identified and synthesized evidence from studies investigating oxytocin’s role in the psychophysiological correlates of emotion recognition across psychopathy dimensions. However, as no such direct studies were identified, we instead compiled and analyzed research examining these variables separately. A scoping review was conducted to capture studies reporting on psychopathy or oxytocin in relation to facial emotion recognition, whether or not they included central or peripheral psychophysiological measurements – retrieving 66 articles. We found distinct emotion recognition outcomes between psychopathy dimensions, some even with opposing neural activity in response to emotional expressions, particularly those of negative valence, as assessed through neuroimaging, electrophysiology, eye-gazing, and pupillometry. Oxytocin presented suggestive positive/compensatory effects on social salience, enhancing emotion recognition, and increasing pupil dilation, and eye-gazing towards faces, and decreasing brain activation towards negative emotions. This review highlights the critical need for future studies to bridge the gap between psychopathy and oxytocin research by exploring their interaction on shared psychophysiological correlates. Such efforts could facilitate the identification of dimension-specific diagnostic biomarkers and targeted interventions for psychopathy.

## Introduction

Societies rely on trust and cooperation, which are essential for maintaining communal, intimate, professional, legal, and commercial relationships. These societal functions are supported by the neurobiological circuitry humans evolved to facilitate prosocial behavior. However, this circuitry may be impaired in individuals with psychopathic traits [1–5], compromising ethical behavior across different economic, political, and social power strata. Such dysfunction can manifest in behaviors ranging from petty crimes to white-collar offenses and even genocides, posing a threat to the safety and well-being of society.

### Psychopathy dimensions and measurement tools

Psychopathy is a personality disorder with concepts and definitions that have evolved since the 1800s [6]. It is primarily characterized by traits such as superficial charm, manipulative behavior, lack of guilt or remorse, and lack of empathy, as well as poor behavior control, unrealistic goals, juvenile delinquency, and criminal versatility [7].

While psychopathy itself is not listed as a diagnosis in the Diagnostic and Statistical Manual of Mental Disorders, 5^th^ edition, text revision (DSM-5-TR), Antisocial Personality Disorder [ASPD] is included [8]. The diagnostic criteria for ASPD encompass a pattern of disregard for and violation of the rights of others, deceitfulness, impulsivity, irritability and aggressiveness, irresponsibility, and lack of remorse. Although deceit and manipulation are also central features of ASPD, the terms “psychopathy” and ASPD refer to distinct constructs. Specifically, traits such as lack of empathy, inflated self-appraisal, and superficial charm are traditionally more characteristic of psychopathy and are not required for an ASPD diagnosis [8]. Nevertheless, the presence of psychopathic traits in individuals with an ASPD diagnosis is a stronger predictor of recidivism in forensic populations [8].

Several psychological instruments are commonly used to assess psychopathic traits; however, the most frequently used in research contexts include: PCL-R (Hare’s Psychopathy Checklist – Revised), PCL:SV (Hare’s Psychopathy Checklist: Screening Version), SRP (Self-Report Psychopathy Scale), TriPM (Triarchic Psychopathy Measure), PPI (Psychopathic Personality Inventory), and LSRP (Levenson’s Self-Report Psychopathy Scale) (see S1 File for further details about psychopathy assessment instruments and their associations with the PCL-R’s factors and facets).

The PCL-R [9], typically administered in an interview format, remains one of the most widely used psychopathy assessment tools worldwide [10]. It consists of 20 items grouped into two factors [9]: Factor 1 (F1), encompassing the Interpersonal and Affective facets, and Factor 2 (F2), comprising the Lifestyle and Antisocial facets [8,11]. The Interpersonal facet evaluates traits like superficiality, grandiosity, and deceitfulness, while the Affective facet assesses lack of remorse, lack of empathy, and failure to accept responsibility. The Lifestyle facet measures impulsivity, lack of long-term goals, and irresponsibility (term used by the authors [12]), whereas the Antisocial facet captures poor behavioral controls, adolescent antisocial behavior, and adult antisocial behavior. Together, these items assess traits associated with psychopathy [12]. Moreover, the diagnostic criteria for ASPD align more closely with the Lifestyle and Antisocial facets of F2 than with those of F1 [8,11].

Some authors propose the existence of two main clusters of psychopathic traits, referred to in this review based on the PCL-R factor distinction: F1-related traits, associated with fearlessness, reduced empathy-related neural and physiological responses, self-control, and manipulative behavior; and F2-related traits, associated with reactive temperament, impulsivity, reward sensitivity, and retaliatory behavior stemming from past traumatic experiences [5,13]. Despite ongoing research, the biological underpinnings of these trait clusters remain poorly understood.

### Social salience and emotion recognition impairments in psychopathy and central and peripheral correlates

Social salience refers to the allocation of attention to socially relevant stimuli. It plays a critical role in emotion recognition and allows for both cognitive and affective empathy, as well as socially adaptive behavior [14,15]. Cognitive empathy is generally defined as the ability to infer others’ perspectives or mental states, including intentions, emotions, and thoughts [16,17], and is typically assessed through explicit emotion recognition paradigms (i.e., tasks requiring the classification of emotions). The literature is mixed on whether psychopathy is associated with deficits in cognitive empathy, and some studies suggest these deficits may depend on individual and contextual factors [18]. However, cognitive empathy deficits have been more frequently associated with ASPD and F2-related traits [18,19], and may require conscious cognitive effort to compensate [20,21]. Affective empathy, on the other hand, is usually defined as sharing another’s emotional experience or mental state [16,17] and is assessed through implicit emotion recognition paradigms (also known as emotion processing). Deficits in affective empathy are generally associated with psychopathy, particularly F1-related traits [18,19].

Social salience and implicit emotion recognition are linked to a network of regions, including the amygdala, ventral striatum, anterior insula, orbitofrontal cortex, and anterior cingulate cortex [14,22,23]. Temporal cortical regions (e.g., fusiform and superior temporal cortex) also participate in facial recognition (rather than inanimate object recognition), with the amygdala and anterior insula being possibly implicated in the recognition of emotional faces rather than neutral faces [24]. Specific emotions, such as fear, sadness, and happiness, seem to elicit heightened amygdala responses, while disgust and anger more strongly activate the anterior insula [24]. Although non-dichotomic models of emotions have emerged, emotions are often described based on their valence: positive valence emotions, generally associated with pleasant experiences (e.g., happiness, surprise), and negative valence emotions, typically related to unpleasant experiences (e.g., fear, sadness, anger) [25].

Effective salience attribution to social stimuli involves directing attention to relevant social cues (e.g., the eyes of a fearful face, the mouth of a happy face) [26] and may be impaired in individuals with psychopathy [27]. This impairment may influence empathic processes and decrease aggression through at least three possible mechanisms [27]: a) empathic inhibition of aggressive impulses in response to others’ distress; b) empathic learning to avoid harmful actions arising from their association with negative valence; and c) making empathic decisions to avoid actions tied to others’ distress.

Focusing on the first process, the cognitive Violence Inhibition Mechanism model posits that low-level amygdala stimulation by aversive stimuli (e.g., fearful, sad, and painful expressions) [24] can initiate freezing via connections to the hypothalamus and periaqueductal gray [28]. Similarly, the Integrated Emotions System model emphasizes amygdala dysfunction in explaining deficits in implicit emotion recognition associated with psychopathy [29], suggesting that the amygdala is crucial for stimulus-reinforcement learning, particularly aversive conditioning [29]. This mechanism allows individuals to associate violent actions with aversive feedback (e.g., victim distress), promoting behavioral avoidance – a process impaired in psychopathy [30]. Additionally, the amygdala is thought to play a key role in generating empathy and approach behaviors toward others’ distress-related emotions, such as fear [31]. The amygdala, along with the striatum, is essential for reinforcement-based learning, while the anterior insula and dorsomedial prefrontal cortices are involved in selecting responses to inhibit based on expected value cues [32]. The maintenance of value representations of response choices also involves the rostromedial, ventromedial frontal, and posterior cingulate cortices [32].

F1-related traits have been associated with hypoactivation of the amygdala and anterior insula in response to distress and pain cues, leading to fearlessness and low emotional reactivity [33]. This hypoactivation may explain the lack of violence inhibition underlying instrumental aggression [5,27]. In contrast, anger has been linked to hyperactivity in the amygdala, hypothalamus, and periaqueductal gray in response to threats, frustration, and social provocations [34]. F2-related traits, therefore, have been associated with hypersensitivity to distress cues and high emotional reactivity, which, when combined with amygdala hyperactivation, may lead to reactive aggression [5,27,33,35]. However, amygdala dysfunction (especially hypoactivation) is not consistently associated with psychopathy across studies, highlighting the need for further research in this area [36–38].

Regarding other nervous system measurements, eye-gazing (i.e., the act of maintaining eye contact with someone, enabling emotion recognition) [39] and pupil dilation when viewing faces have been closely related to social salience attribution within facial emotion recognition paradigms, a skill often impaired in psychopathy [40–44]. Although eye-gazing also involves the peripheral nervous system [45], it is actively controlled by brain structures [46, 47] and is thus referred to here as a central nervous system correlate. On the other hand, pupil dilation is a common positive index of autonomic nervous system activity, associated with emotional arousal, which increases with the allocation of attentional resources to a stimulus [48,49] and with cognitive load [50]. Pupil dilation has also been associated with recognizing emotional authenticity [51]. Salience attribution and facial emotion recognition are essential for generating appropriate responses to others [27,32], enhancing social adaptation [52].

### Oxytocin’s role in social salience and emotion recognition and central and peripheral correlates

Oxytocin (OT) is a neuropeptide released from the paraventricular and supraoptic nuclei of the hypothalamus into both the central and peripheral nervous system [53]. OT receptors are widely expressed across the human brain, predominantly in regions such as the amygdala, anterior cingulate cortex, piriform cortex, hypothalamus, nucleus of the solitary tract, and hypoglossal nucleus [54,55]. These areas are associated with fear responses, motivation and decision-making, sexual and parenting behavior, olfactory and gustatory processing, breathing, and eye movement [54]. In addition, neurons transporting OT establish connections with the prefrontal cortex, sensory cortices, and hippocampus, supporting the regulation of complex behaviors, social interactions, and emotions, as well as processing of sensory cues, and memory [53,55].

In the periphery, OT receptors are found in several systems and organs, including the cardiovascular and reproductive systems [55]. By mediating smooth muscle contractions in the heart, breast, and uterus, OT plays a crucial role in lactation, parturition, orgasm, and cardiovascular regulation [55]. Studies exploring OT gene expression patterns across the human brain have identified enriched expression in a broad range of subcortical regions, namely in the basal ganglia and olfactory regions [56]. These regions are involved in the regulation of cognitive states, including aversive (e.g., anxiety, stress, fear, unpleasant), anticipatory (e.g., reward, anticipation, motivation, learning), appetitive (e.g., sexual, taste, seeking), and both appetitive/aversive (e.g., facial expressions and emotions of positive or negative valence) states [56]. Notably, OT gene expression in these brain areas appears more closely related to the processing of social stimuli than non-social ones, underscoring OT’s critical role in emotion processing and social adaptation [56]. In accordance with its central and peripheral targets and its gene network, several studies have highlighted the essential role of OT in reproduction, learning, stress regulation, adaptation, attachment, social touch, and bonding – all vital for survival in humans and other mammals [54,57,58].

OT’s effects have also been extensively studied through intranasal administration. A single dose of intranasal OT (in-OT) has been shown to enhance both emotional and cognitive empathy abilities in non-clinical samples, particularly in tasks involving emotion recognition [59]. Meta-analyses further confirm a positive effect of in-OT in emotion recognition in non-clinical populations [60,61]. Along with evidence of increased trust, social bonding, and cooperation following in-OT administration [62–64], these findings reinforce OT’s role in promoting social adaptation.

Furthermore, in-OT modulates brain activity, reducing amygdala responses to aversive stimuli and influencing regions such as the temporal lobes, anterior cingulate cortex, and insula [65,66]. In-OT also increases eye-gazing toward emotional and neutral faces [67] and enhances pupil dilation in response to emotional or social stimuli [52,68]. In addition, in-OT has been found to increase N170 amplitude [69], an event-related potential associated with facial structural encoding, which typically occurs between 130–200 ms after stimulus perception [70]. Together, these neural and psychophysiological changes support OT’s involvement in empathy, emotion recognition, and social salience.

To further clarify OT’s mechanisms in social behavior, the “Social Adaptation Model of OT Function” was proposed [71]. According to this model, OT reduces negative affect by dampening the emotional reactivity network (e.g., amygdala, anterior cingulate cortex, anterior insula) while upregulating the emotion regulation network (e.g., medial prefrontal cortex, ventrolateral prefrontal cortex, dorsolateral prefrontal cortex). This regulation facilitates decreased threat sensitivity and promotes perceptions of a safer environment, enhancing trust and prosocial behavior [72]. Some studies also suggest that OT improves fear differentiation, reducing fear in nonspecific threat situations while amplifying fear responses when faced with immediate and predictable threats [73].

Additionally, the model posits that OT activates the saliency network (e.g., ventral tegmental area, amygdala, anterior insula, superior temporal sulcus), thereby enhancing the attribution of salience to social stimuli and promoting more adaptive emotional responses to others [71]. This framework aligns with the “Social Salience Hypothesis of OT”, which proposes that OT may enhance social salience, and, as a consequence, empathy, stress attenuation, and prosocial behaviors toward “in-group” members [26]. Some authors argue that OT’s modulation of amygdala activity reflects an adaptive balance between bottom-up and top-down attention systems, influenced by context, sex, personality traits, and degree of psychopathology [26,74].

Given these findings, OT’s potential to promote healthy and adaptive social behaviors appears promising, although heterogeneous results in clinical samples characterized by emotion dysregulation and social impairments. In clinical studies, OT has normalized hyperactive or hypoactive brain activity, facilitating social interactions, reducing withdrawal, and enhancing attention to social cues in individuals with autism spectrum disorder (ASD), social anxiety disorder (SAD), schizophrenia, borderline personality disorder (BPD), and posttraumatic stress disorder (PTSD) [71]. However, other studies have found non-significant effects of OT on emotion recognition in mixed clinical samples (e.g., PTSD, BPD, ASD) [60], or no improvements in negative symptoms in schizophrenia (i.e., amotivation and diminished expression) [75].

Variations in OT system function may partly explain these discrepancies. Some studies report lower OT concentration in individuals with BPD and schizophrenia [76], especially associated with negative symptoms of schizophrenia and higher adverse childhood experiences [77]. In contrast, higher OT levels have been observed in individuals with bipolar disorder and obsessive-compulsive disorder [76]. For ASD or SAD, OT concentrations in adults do not consistently differ from those in healthy controls [76], although lower OT levels have been reported in children with ASD [78] and females with high anxiety levels [79]. These findings underscore the need for further studies exploring OT effects across specific clinical groups, taking into account variables such as sex, in-OT dosage, and personality traits [59,64,75].

### Oxytocin and impairments in social salience and emotion recognition in psychopathy

Taking into account the findings previously mentioned, a parallel between psychopathy’s social impairments and the role of OT system in social adaptation may be outlined, along with associated central and peripheral correlates. For instance, psychopathy-related impairments across central (e.g., amygdala, insula, hypothalamus) [5,33] and peripheral (e.g., eye-gaze, pupil dilation) correlates [40–44] of social salience and emotion recognition overlap with brain regions enriched with OT receptors and functions modulated by OT [52,65–67,69]. Consistently, psychopathy is characterized by deficits in empathy, manipulative behavior, social norms’ disruption, and aggression – leading to social dysfunction [7,8]. In parallel, in-OT administration shows positive effects on empathy, prosocial behavior, and cooperation [59,62–64], thus promoting social adaptation in non-clinical and some clinical populations [71].

Two studies involving adult populations with ASPD further support the role of OT in normalizing implicit emotion recognition deficits, regardless of whether these deficits involve hyperactivation or hypoactivation. In one study, individuals with ASPD (both males and females) presented right amygdala hyperactivation in response to angry faces (compared with individuals without ASPD), but not to fearful or happy faces [80]. This hyperactivation was reversed following a 24 international units (IU) dose of in-OT. Although some participants met the criteria for psychopathy, psychopathic traits were not specifically analyzed. Nevertheless, this study supports a role of OT in decreasing neural hyperactivation in response to anger, thereby contributing to reducing reactive aggression [80], typically associated with ASPD and F2-related traits [5].

In the second study with violent male offenders, individuals with both ASPD and psychopathy (compared with those with ASPD without psychopathy) displayed a reduced response to facial fearfulness intensity in the bilateral mid-cingulate cortex, which was counteracted by a 40 IU dose of in-OT [81]. Participants with both ASPD and psychopathy presented higher proactive, reactive, and total aggression scores, and higher scores on all PCL-R facets. Hypoactivation in the mid-cingulate cortex, essential for integrating fear cues, reinforces the lack of empathy and contributes to increased aggression, which in-OT appears to normalize [81].

A recent study explored brain modulation under in-OT in children with conduct problems, some presenting high callous-unemotional (CU) traits (precursors of F1-related traits) [82]. A 24 IU dose of in-OT had no effects on amygdala activity in response to fearful expressions but increased the activity in the posterior cingulate cortex/precuneus in response to happy faces. Although these results support the OT role in normalizing activation in areas related to cognitive and affective empathy, the lack of effects on the amygdala may reflect limited sample size and insufficient statistical power to detect subgroup differences based on CU traits.

Regarding explicit emotion recognition deficits, one study administered a single 24 IU dose of in-OT to adult forensic patients with ASPD [83]. Although psychopathic traits were not assessed, OT administration reversed significant deficits in recognizing fearful and happy faces [83]. Similarly, in residential youths with conduct problems, a 24 IU dose of in-OT improved empathy toward emotional film clips among youths with high CU traits [84]. An overall positive effect on accuracy was also observed, specifically in recognizing fearful faces [84]. These findings stress the role of OT in normalizing emotion recognition deficits among individuals with ASPD (F2-related traits) and enhancing empathy among those with CU traits (F1-related), consistent with theoretical distinctions between cognitive and affective empathy.

Other behavioral paradigms provide additional support. In a gaze aversion task assessing reactive social dominance, a forensic sample of males with psychopathy and controls were compared under in-OT administration [85]. This task assesses gaze aversion of masked angry, happy, and neutral faces. It is considered a potential measure of reactive social dominance, by linking slower gaze aversion from angry faces (vs. happy faces) with higher reactive dominance, and faster gaze aversion from angry faces with submissiveness [85]. Higher reactive dominance was observed for Interpersonal (F1-related) and Antisocial (F2-related) PCL-R facets, with 24 IU in-OT decreasing this heightened dominance. Although individuals with psychopathy did not differ from controls in gaze aversion latency, these results suggest a potential role of OT in mitigating reactive dominance behaviors.

In relation to endogenous OT levels, one study among male forensic patients with ASPD found that higher urinary OT levels were associated with PCL-R’s F2 scores [86]. Another study also reported lower salivary OT levels in forensic patients compared to healthy controls, with higher psychopathy scores – particularly F2 – correlating with higher urinary OT levels [87]. These findings suggest that elevated OT levels in individuals with high F2 traits might reflect a compensatory anxiolytic response to heightened emotional reactivity and anxiety, possibly influenced by the prison environment [86,87].

In residential youths, lower salivary OT levels were associated with higher severity of conduct problems (F2-related), with these youths being more prone to present CU traits (F1-related) [88]. Furthermore, youths with high CU traits and low emotional neglect (associated with F1-related traits or “primary psychopathy” as denominated by the authors) presented lower salivary OT levels compared with those with high CU traits and high emotional neglect (associated with F2-related traits or “secondary psychopathy”) [89].

Although the literature remains scarce, emerging evidence supports a possible role of OT in the distinct constellation of traits and neurocorrelates inherent to F1 and F2 psychopathy dimensions – often in opposing directions. These findings suggest different OT profiles and effects, with OT enhancing social salience among individuals with F1-related traits, who also seem to present lower OT levels. On the other hand, OT seems to dampen reactivity to aversive stimuli among those with F2-related traits, who may present higher OT levels. Both pathways could contribute to social adaptation, by improving cognitive and affective empathy and reducing aggression – whether proactive or reactive. Moreover, most relevant studies employed emotion recognition paradigms, supporting their utility for future research.

Despite most studies focused on samples with ASPD or youths with conduct problems and/or CU traits, no review has specifically addressed dimension-specific research on OT function and the neurocorrelates of psychopathic traits across both the central and peripheral nervous systems. Previous reviews on psychopathy impairments [2,5,33] or OT effects [90–92] have focused on some of these aspects independently, often neglecting the autonomous nervous system. Furthermore, to our knowledge, no review or meta-analysis has specifically addressed the role of OT across distinct psychopathy dimensions. For instance, one single review examined the role of OT in ASPD [93], yet this DSM [8] diagnosis does not encompass the full range of psychopathic traits. Similarly, while a meta-analysis explored the effects of OT on empathy-related tasks and questionnaires, it discussed the implications for psychopathy research without systematically reviewing the literature [59].

Understanding the causal and biological mechanisms underlying psychopathic traits is crucial for individuals affected by the condition, their families, victims of abuse, and society as a whole. Advancing our understanding in this area could improve neuroscientific models of social cognition, facilitating better prevention, screening, diagnosis, and treatment strategies for psychopathy. Potential outcomes include the development of: (i) psychopathy biomarkers – less prone to manipulation than self-reported measures – that could serve as screening tools for professions requiring ethical integrity, such as politics, executive roles, judiciary, or law enforcement; and (ii) therapeutic interventions or medications aimed at normalizing neuromodulators implicated in psychopathy. Consequently, conducting a review on the connection between OT and psychopathy regarding facial emotion recognition correlates would significantly contribute to consolidating knowledge, guiding future research.

### Aims

Given the psychopathy-related social and neural impairments mentioned above, along with the overlapping behavioral and neural effects of in-OT in humans, we aimed to conduct a scoping review to address the following question: What evidence exists on the effect of OT on behavioral, central, and peripheral nervous system correlates of facial emotion recognition across distinct psychopathy dimensions, analyzed as a continuum (rather than through categorical diagnoses), and in relation to PCL-R factors or related traits (i.e., F1- and F2-related traits) assessed with comparable instruments)?

For conciseness and to warrant comparability across findings, we focused on emotion recognition paradigms. In particular, we aimed to analyze explicit facial emotion recognition tasks (i.e., those requiring participants to categorize emotions) and implicit facial emotion recognition tasks (i.e., those involving the mere visualizing of emotional expressions).

It can be hypothesized that: 1) the OT system may be abnormal in individuals with psychopathic traits, particularly concerning emotion recognition correlates; and 2) OT-based interventions (pharmacological or behavioral) could be promising in addressing these deficits. However, to date, there is a lack of studies examining behavioral, central, and peripheral nervous system correlates of psychopathic traits (analyzed as a continuum in adults), considering the specificities of each psychopathy dimension and OT’s role in these neurocorrelates. Consequently, our main objective was to systematize the literature addressing the above research question, discuss the findings in an integrated manner, and identify patterns and gaps in knowledge to guide future studies on psychopathy and/or the OT system.

## Methods

### Search strategy

A systematic search strategy was conducted following the Preferred Reporting Items for the Systematic Reviews and Meta-Analyses (PRISMA) statement [94] and the PRISMA Extension for Scoping Reviews (PRISMA-ScR) guidelines [95]. This scoping review was registered on OSF Home (Registration DOI: https://doi.org/10.17605/OSF.IO/6SYD5).

The search was carried out on August 29, 2024, in the following databases: PubMed (NLM), Web of Science Core Collection (Clarivate), and PsycINFO (EBSCOhost).

Initially, to gather evidence on OT’s role in emotion recognition correlates across distinct psychopathy dimensions (i.e., F1- and F2-related traits), a broad search equation was built to identify studies involving psychopathy, OT, and emotion recognition, without specifying behavioral, central, and/or peripheral correlates. The search equation was as follows: “(psychopathy OR psychopathic) AND oxytocin AND (facial OR face) AND (emotion recognition OR passive viewing) AND adult.” However, no articles captured by this search met the inclusion criteria.

As a result, we refined our original research question into two sub-questions: 1) What behavioral and/or neurocorrelates of emotion recognition have been identified across distinct psychopathy dimensions?; and 2) What evidence exists regarding OT’s role in those correlates, irrespective of psychopathy?

For each sub-question, the following search terms were used: 1) for psychopathy studies: psychopathy, psychopathic, emotion recognition, passive viewing, facial, face, adult, “functional magnetic resonance,” fMRI, EEG, electroencephalography, ERP, evoked potentials, neuroimaging, eye-tracking, eye-gaze, saccade, and pupil*; 2) for OT studies: oxytocin, emotion recognition, passive viewing, facial, face, adult, “functional magnetic resonance,” fMRI, EEG, electroencephalography, ERP, evoked potentials, neuroimaging, eye-tracking, eye-gaze, saccade, and pupil*. The complete search queries are available in S1 Table.

**Fig 1:**
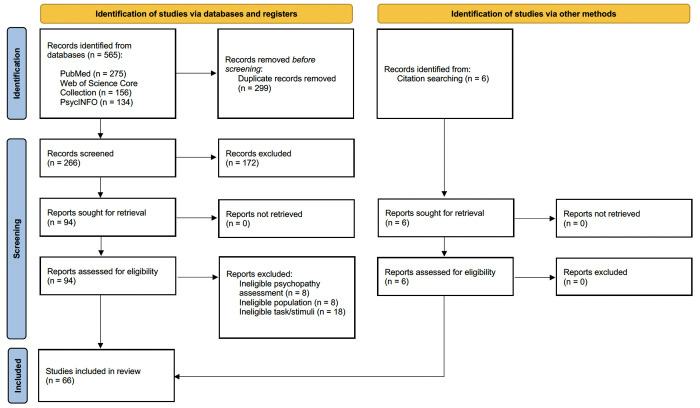
Flow diagram of the screening process.

All articles retrieved were gathered in the Covidence systematic review software (Veritas Health Innovation, Melbourne, Australia, 2014). Duplicates were removed, and two independent researchers (SFN and FF) conducted the selection and data extraction (Fig 1). Article selection was based on abstract and full-text reviews according to pre-specified eligibility criteria. Any disagreements were resolved by a third researcher (DP).

Articles meeting all inclusion criteria were then exported to EndNote^TM^ (Endnote 20, Clarivate, Philadelphia, 2013) for analysis. The reference lists of these articles were also reviewed to capture relevant studies not identified through the initial search strategy. A total of 66 studies published between 2004 and 2023 were included.

### Eligibility criteria

For this scoping review, studies were selected based on the following inclusion criteria: i) an explicit or implicit facial emotion recognition paradigm/task was used (i.e., participants were either asked to identify the facial emotion displayed or were not explicitly instructed to do so, respectively); ii) in psychopathy-related studies, psychopathic traits were assessed in healthy and/or ASPD populations, measured and analyzed as a continuous variable with a validated instrument (e.g., PCL-R, PCL-SV, SRP, TriPM, PPI, or LSRP), regardless of whether participants met the criteria for classification as a psychopath; iii) inclusion of adult (≥ 18 years old) male and/or female samples.

The exclusion criteria were as follows: i) tasks involving only images of eyes or mouths, rather than whole faces; ii) studies focusing on clinical populations other than ASPD, psychopathy, or forensic psychiatric patients; iii) studies related to OT where OT was not measured or administered; iv) genetic-only studies; v) animal studies; vi) conference papers; vii) reviews; viii) meta-analysis; ix) posters; x) dissertations; xi) articles not published in peer-reviewed journals; and xii) articles published in languages other than English, Spanish, or Portuguese.

### Quality assessment

Each study was appraised using a 12-criteria scoring system (see S2 Table for the full list), where each criterion was rated on a scale of 0–3. Scores reflected the extent to which each criterion was addressed in the article, ranging from absent (0) to little (1), some (2), or strong (3) evidence, based on an appraisal method from a previous review [58]. The 12 criteria were as follows: i) clearly stated hypothesis and objectives; ii) use of methods capable of testing the hypothesis; iii) clearly described recruitment procedures and eligibility criteria for participants; iv) efforts to account for demographic or neuropsychological variability among participants; v) inclusion of an a priori power calculation to define sample sizes; vi) use of valid and clearly described cognitive tasks assigned to elicit the desired effects in participants; vii) valid and clearly described psychopathy measurement tools, when applied; viii) adequate and clearly described methods for OT administration or measurement, when performed; ix) clear description of techniques, parameters, and preprocessing in studies assessing central and/or peripheral nervous system activity; x) statistical methodology clearly explained; xi) use of a consensual statistical significance threshold; and xii) reporting of effect sizes for significant results.

The overall quality of each study was calculated by dividing the total score across the 12 criteria by the maximum possible score applicable to the study. Quality scores were categorized as “low” (≤ 65%), “medium” (65–85%), or “high” (≥ 85%). No study was excluded based on its overall quality score.

## Results

**Fig 2:**
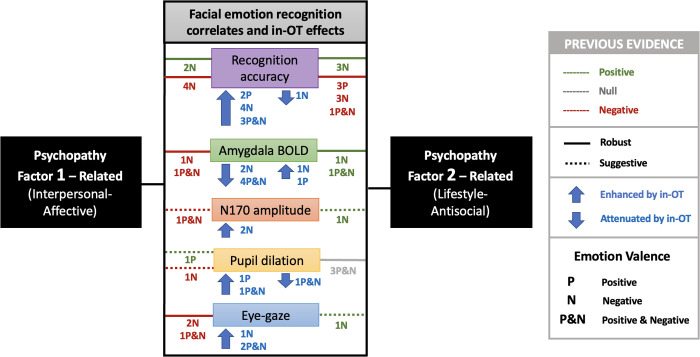
Graphical summary of the main findings related to emotion recognition, central and peripheral functional correlates for each psychopathy dimension, and the effects of intranasal oxytocin (in-OT) on these correlates. The figure illustrates the distinct – and often opposing – behavioral deficits and patterns of central and peripheral nervous system activity (particularly amygdala BOLD activation, N170 amplitude, pupil dilation, and eye-gazing) associated with psychopathy’s Factor 1 (F1) and Factor 2 (F2) related traits. Specifically, for negative valence emotions, F1-related traits often exhibit greater deficits in recognition and central and peripheral hypoactivity, whereas F2-related traits present higher recognition accuracy and central and peripheral hyperactivity. IN-OT robustly improved recognition accuracy and enhanced both central and peripheral activity, particularly in response to negative emotions, while attenuating amygdala reactivity. This figure includes only the results related to associations with each psychopathy dimension individually. It does not present associations related to the total psychopathy score or subscales that overlap both dimensions. Both implicit and explicit facial emotion recognition studies are considered. Lines in the figure represent associations reported in previous studies and are colored according to their direction (positive, null, or negative), while adopting a pattern to indicate the strength of the evidence (robust or suggestive). Solid lines indicate robust findings (replicated results), whereas dotted lines indicate suggestive findings (not replicated). The letters “P,” “N,” and “P&N” correspond to the emotional valence of the stimuli (positive, negative, or both), and the numbers preceding the letters indicate the number of studies reporting each finding.

A graphical summary of the main findings from this review is presented in Fig 2. A detailed description of the characteristics of all the studies included in the review is provided in S3 and S4 Tables.

### Psychopathy, oxytocin, and facial emotional recognition

This scoping review found no studies that assessed the effect of in-OT on psychopathic traits analyzed dimensionally, and using a facial emotion recognition paradigm in adults.

### Psychopathy and facial emotion recognition

#### Behavior.

Among the 33 studies exploring the relationship between psychopathy and facial emotion recognition accuracy, 27 involved explicit tasks and six used implicit tasks. Thirteen studies (39.4%) reported lower recognition accuracy associated with psychopathy (total scores and/or subscales), with most conducted in forensic rather than community samples.

For psychopathy’s total score, lower explicit recognition accuracy was reported for a range of emotional expressions, including angry [96,97], happy [96,97], sad [98–100], disgusting [97,101], and fearful faces [41,100,102,103]. One study found lower accuracy across a broad spectrum – upright and inverted disgustful, fearful, happy, surprised, and composite happy faces [96] – while another observed lower accuracy regardless of valence (angry, fearful, surprised, sad, happy, and disgustful faces) [104]. In addition, slower response times for neutral faces were also reported [105].

Specific facets and subscales were associated with emotion recognition impairments. The Antisocial facet (F2-related) was associated with lower accuracy for happy faces [99] and longer response times for angry faces [106]. The Disinhibition subscale (F2-related) was associated with poorer recognition of disgustful faces [107], and the Meanness subscale (F1- and F2-related) with worse accuracy for fearful faces [107,108].

Two studies reported lower accuracy during implicit recognition tasks. These difficulties were associated with the Callous Affect (F1-related) and Antisocial Behavior (F2-related) subscales, when classifying fearful faces as either neutral or emotional [102], and with the Coldheartedness subscale (unrelated to either F1 or F2) in a task requiring localization of fearful faces [109].

Conversely, two studies (6.67%) reported better emotion recognition accuracy associated with psychopathy’s total score. One study identified enhanced recognition across several emotional expressions (e.g., happy, sad, angry, fearful, surprised, disgustful, and distressed faces) [110], while another found improved accuracy specifically for fearful faces [111].

Six studies (20%) reported mixed results. Although one study found better recognition of sad faces associated with psychopathy’s total score [41], it was also observed worse recognition for sad, happy, and fearful faces [41,100].

At the subscale level, findings varied by facet. The Affective facet (F1-related) was associated with both better recognition of fearful faces [112] and worse recognition of fearful [43,113], disgustful [113], and neutral faces [114]. The Interpersonal facet (F1-related) was associated with better recognition of angry faces [113], but worse recognition of disgustful expressions in female faces [114]. The Lifestyle facet (F2-related) was linked to both enhanced recognition of disgustful [114] and fearful faces [43], and impaired recognition of happy [112] and surprise faces [113]. The Antisocial facet (F2-related) was similarly associated with both improved (disgustful [114], sad, and fearful faces [100]) and reduced recognition (fearful, disgustful, and surprised faces [113]) accuracy.

The remaining 12 studies (36.4%) found non-significant associations between psychopathy (total score or subscales) and emotion recognition accuracy or response time [42,44,115–124].

#### Functional magnetic resonance imaging.

Seven studies employed functional magnetic resonance imaging (fMRI) to examine facial emotion recognition, with four studies using implicit tasks and three using explicit tasks. Most were carried out in forensic rather than community samples. Six studies (85.7%) showed reduced brain activation associated with psychopathy (total score and/or subscales).

One study [125] identified widespread reductions in brain activation during the recognition of emotional faces associated with both psychopathy’s F1 and F2. For happy faces, reduced activity was observed in the bilateral fusiform, right inferior and middle frontal gyri, right orbitofrontal, right dorsomedial prefrontal cortices, and left inferior temporal pole. For fearful faces, reductions were found in the bilateral middle occipital, right inferior frontal, and right supramarginal gyri. Sad faces were associated with reduced activity in the left posterior superior temporal sulcus, right inferior frontal gyrus, bilateral dorsomedial prefrontal cortex, and right supplementary motor area. For painful faces, reduced activity emerged in the middle cingulate, dorsomedial prefrontal cortices, inferior frontal, and left angular gyrus.

Further analysis in the same study [125] revealed distinct activation patterns by psychopathy factor. F1 traits were associated with reduced activation for happy faces in the right middle occipital and bilateral inferior frontal gyri, right ventromedial prefrontal and left orbitofrontal cortices, and right inferior temporal pole; for fearful faces, in the left insula, right ventromedial prefrontal and orbitofrontal cortices, and left supplementary motor area; and for painful faces in the left inferior frontal gyrus and left posterior superior temporal sulcus. F2 traits were linked to reduced response for happy faces in the right supramarginal gyrus and right supplementary motor area; for fearful faces in the right insula, left inferior and middle frontal gyri, and left supplementary motor area; for sad faces in the left fusiform and inferior frontal gyri, and inferior pole; and for painful faces in the right superior temporal sulcus, dorsal anterior cingulate cortex, and striatum [125].

Other studies similarly reported reduced activation associated with psychopathy’s total score. These included lower responses for fearful faces in the bilateral fusiform [119,125] and right middle frontal gyri, dorsomedial prefrontal cortex, inferior temporal pole [125], bilateral cerebellum, and left postcentral gyri [119]; for happy faces, in the right fusiform, left precentral and left lingual gyri, and left cerebellum [119]; and across multiple expressions in the right fusiform gyrus and amygdala (happy, angry, fearful, and neutral faces) [121], as well as in the medial prefrontal cortex (angry, fearful, sad and joyful faces) [120].

At the subscale level, the Emotional Interpersonal subscale (F1-related) was associated with reduced activation in the right inferior frontal cortex, right amygdala, and medial prefrontal cortex for a range of emotional expressions (angry, fearful, sad, and joyful faces) [120], while the Interpersonal facet (F1-related) was associated with reduced activation in the right dorsal amygdala specifically for fearful faces [126]. F2 traits were also associated with lower activity in the frontoparietal cortex, visual areas, and diencephalic-mesencephalic structures, regardless of emotion type (fearful, happy, and neutral faces) [118].

Despite predominantly reduced activation findings, five of the studies (71.4%) also reported regions of increased activation, and one additional study (14.3%) reported solely enhanced responses.

F1 and F2 were associated with higher activation in the anterior insula during painful face processing [125]. F1 alone was associated with left postcentral and right precentral gyri for painful faces, right insula for fearful faces, and left anterior insula and left middle cingulate gyrus for sad faces [125]. Another study reported higher activation in the frontal cortex related to F1 for emotional faces regardless of emotion type (happy, fearful, and angry) [118].

F2-related subscales were also linked to enhanced activity. The Social Deviance subscale was associated with increased right amygdala activation across emotional expressions (angry, fearful, sad, and joyful faces) [120], and the Lifestyle facet was associated with heightened right dorsal amygdala response specifically for angry faces [126], as well as higher dorsomedial prefrontal cortex volume in association with overall emotional faces (happy, surprised, fearful, sad, disgustful and angry faces) [100].

At the whole-score level, psychopathy was positively correlated with activation in the right insula and precuneus during fearful face processing, and with increased activity in the right cerebellum, bilateral fusiform, middle occipital, anterior cingulate, and medial frontal gyri, right precuneus, and left superior parietal lobule for the same emotion [119].

One study further reported associations between better emotion recognition and greater volume among individuals with psychopathy. Specifically, better recognition for sad faces was associated with increased dorsomedial prefrontal cortex volume; for happy faces, higher volume was found in the middle anterior cingulate cortex, anterior insula, inferior frontal gyrus, orbitofrontal cortex, and anterior cerebellum; and for fearful faces, in the somatosensory cortex [100].

#### Electroencephalography.

Four studies used electroencephalography (EEG) to explore the neural correlates of emotion recognition in relation to psychopathy, employing either implicit (one study) or explicit (three studies) tasks. Forensic and community samples were equally represented.

Two studies (50%) reported significant associations between psychopathy and reduced event-related potential (ERP) amplitudes. One study found lower error-related negativity (ERN) regardless of emotional expression (angry and fearful faces) associated with psychopathy’s total score [122]. Another study reported lower N2 amplitude for angry and fearful faces, specifically associated with F2 [127].

The remaining two studies (50%) yielded mixed results concerning ERP components and psychopathy subscales. One study found that the Fearless Dominance subscale (F1-related) was associated with lower N170 amplitude across multiple emotional expressions (angry, fearful, happy, neutral, and calm faces), while the Coldheartedness subscale (neither F1- nor F2-related) was associated with higher N170 amplitude in response to fearful and happy faces [128]. Another study linked the Meanness subscale (F1- and F2- related) to lower N170 and P200 amplitudes for fearful faces, as well as lower late positive potential (LPP) amplitude for fearful and sad faces. This subscale was also associated with poorer recognition of middle-intensity fearful faces. In contrast, the Disinhibition subscale (F2-related) was associated with higher N170 amplitude in response to fearful faces [129].

One of the aforementioned studies also analyzed N170, P3, and LPP amplitudes but found non-significant associations with psychopathic traits [127].

#### Eye-gazing.

Five studies analyzed the relationship between psychopathy and eye-gazing patterns, specifically focusing on dwell time and/or fixations on the eyes during explicit emotion recognition tasks. Most of these studies were conducted in forensic samples.

Three studies (60%) reported that higher psychopathy’s total scores were associated with reduced dwell time and/or fewer fixations on the eyes, regardless of emotional valence (angry, disgustful, happy, fearful, sad, surprised, and neutral faces) [44]. Reduced fixations on the eye region were also observed in association with specific psychopathy subscales: the Boldness subscale (F1-related) in response to multiple emotional faces (angry, disgustful, fearful, happy, sad, and surprised faces) [42]; and the Primary Psychopathy scale (F1-related) in response to fearful and angry faces [123].

One study (20%) found opposing effects between psychopathy factors. Lower fixations on the eyes of fearful faces were associated with the Interpersonal facet (F1-related) and psychopathy’s total score, while higher fixations were linked to the Antisocial facet (F2-related) [43]. Aligned with this, worse recognition accuracy for fearful faces was observed for the Affective facet (F1-related), and better accuracy was associated with the Lifestyle facet (F2-related) [43].

One study (20%) found non-significant associations between psychopathic traits and fixations on facial features [108].

#### Pupillometry.

Three studies investigated the association between pupil dilation and psychopathy during emotion recognition tasks, implicit (two studies) or explicit (one study), predominantly carried out in forensic samples.

The only study using an explicit emotion recognition task (33.3%) reported that a lower pupil dilation for fearful, happy, and sad faces was associated with the Meanness subscale (F1- and F2-related). This study also found an association between this subscale and worse recognition accuracy for fearful faces and better for sad faces [41].

Another study (33.3%) found that lower pupil dilation to angry faces was associated with the Interpersonal facet (F1-related), while higher pupil dilation for happy faces was linked to both the Affective and Interpersonal facets (F1-related) [40].

The remaining study (33.3%) found non-significant associations between psychopathy’s total score and pupil responses across facial expressions (fearful, happy, neutral, disgustful, angry, and sad faces) [130].

### Oxytocin and facial emotion recognition

#### Behavior.

A total of 19 studies analyzed the effects of in-OT administration on emotion recognition. Most studies administered a 24 IU dose (14 studies), while others used 20 IU (one study), 40 IU (one study), 12, 24 or 48 IU (one study), and 8 or 24 IU alongside 1 IU of intravenous OT (iv-OT) (two studies). Seventeen studies examined explicit emotion recognition, and two focused on implicit tasks. None included psychopathy assessments.

Ten studies (52.6%) reported improved emotion recognition under in-OT compared with placebo (PL). Among explicit emotion recognition tasks, improvements were observed for fearful [131,132], happy [133,134], and angry faces [133,134]. Two studies reported reduced recognition thresholds for angry faces [132,135], and one found that in-OT reduced emotional bias in the recognition of neutral faces [136]. Another study observed improved recognition accuracy for disgustful faces in the context of an angry body under OT [137].

Two studies revealed improvements in explicit recognition tasks regardless of emotional valence. One found enhanced recognition across emotions (angry, disgusted, fearful, happy, sad, and neutral faces), specifically among older males [138], while another observed overall improvements, particularly for happy faces (vs. angry) [139].

Concerning implicit recognition, one study using an emotional gaze cueing task found that in-OT increased gaze-cued attentional orientation happy and fearful faces but not for neutral ones [140].

In contrast, two studies (10.5%) reported worse performance under in-OT: one identified a positive bias in recognizing negative emotions [141]; and another observed delayed responses for happy faces [52]. One additional study (5.26%) reported mixed results: slower recognition of fearful faces under in-PT, but decreased misclassification rates of surprised and neutral faces [142].

Six studies (31.6%) found non-significant effects of in-OT on accuracy, response time, or emotional ratings. These included five explicit recognition studies and one that employed an implicit task [67,143–147].

One additional study, which did not involve in-OT but measured salivary OT levels in primiparous mothers after breastfeeding or infant holding, found that higher endogenous OT levels were associated with improved recognition of happy (vs. angry) and angry (vs. neutral) faces [148].

#### Functional magnetic resonance imaging.

Six fMRI studies examined the effects of in-OT on brain activation during emotion recognition tasks. Four studies administered 24 IU, one used 12 IU, 24 IU, or 48 IU, and another employed 8 IU, 48 IU, or 1 IU of iv-OT. Three studies used explicit tasks, and two used implicit ones.

Five studies (83.3%) presented significant attenuation of amygdala activity under in-OT (vs. PL). This attenuation was observed: in the right amygdala, across emotions (happy, fearful, angry, and neutral faces in one study, and angry, happy, and neutral in another) [144,149], and particularly to fearful faces (vs. happy) in a masked eyes condition [143]; in the left amygdala, in response to fearful faces, which was moderated by fear intensity [136]; and bilaterally, in response to facial features – the eyes (vs. mouth) of angry faces and the mouth (vs. eyes) happy faces in a masked condition [150]. The same study found reduced functional connectivity between the left amygdala and the left fusiform gyrus in response to the eyes of angry faces (vs. neutral) [150].

Several studies reported broader brain activation effects of in-OT. One study observed decreased activation in the anterior cingulate cortex and left mid-temporal gyrus for fearful faces (vs. happy) [143]. Another study found region-specific reductions depending on stimulus type and duration [150]. For the eyes of angry faces (vs. mouth): in the inferior occipital cortex, temporal ventral stream, and brainstem during short presentations; and in the mid-temporal gyrus, superior colliculi, striatum, and left fusiform gyrus for both short and long stimulus presentations. Additionally, decreased activation in the medial superior frontal gyrus was noted in response to the mouth of happy faces (vs. eyes) [150].

One study involving nulliparous women found no overall effect of in-OT (vs. PL) on brain activation in response to crying faces [151]. However, subgroup analyses revealed that: in women with attachment anxiety, OT decreased activation in the right amygdala and bilateral insula. In contrast, women with attachment avoidance presented increased activation in the bilateral amygdala and left inferior orbitofrontal cortex.

#### Electroencephalography.

Three studies explored the effects of in-OT (24 IU and 60 IU) on neural dynamics during emotion recognition tasks, including two implicit and one explicit task.

In two studies (66.7%), OT (vs. PL) increased N170 amplitude: one reported this effect for infant (vs. adult) and sad (vs. happy) faces in mothers [145]; and the other for fearful faces (vs. neutral) [69]. The latter study also found reduced N170 latency for fearful faces (vs. neutral) and for the eyes (vs. mouth) of neutral faces [69]. Moreover, LPP amplitude increased during the processing of infant (vs. adult) faces in mothers. Non-significant effects were observed for P100 and early posterior negativity (EPN) amplitude and latency [69].

The third study (33.3%) found non-significant OT effects in neural sensitivity in the occipitotemporal and medial-occipital regions, as measured by frequency-tagging EEG [147].

#### Eye-gazing.

Two studies explored the effects of in-OT administration (24 IU), compared with PL, on eye-gazing during the explicit recognition of emotional faces.

Across these studies, OT increased dwell time on the eyes of emotional faces. One study found this effect to be specific to sad faces presented at lower intensity levels [132]. Another study reported enhanced dwell time on the eyes of happy and angry faces, but only during the early exploration phase [52]. Despite this attentional modulation, the same study study observed slower responses to happy faces and non-significant effects on recognition accuracy.

Complementing these findings, a third study applied a dot-probe paradigm and showed that in-OT facilitated attentional shifts towards happy faces, although this effect did not translate into differences in probe detection rates [152].

#### Pupillometry.

Three studies explored the effects of in-OT administration (two using 24 IU and one using 40 IU), compared with PL, on pupil dilation during the explicit recognition of emotional faces.

In two of these studies (66.7%), in-OT increased pupil dilation in response to emotional faces. One study observed this effect specifically for happy and male (vs. female) faces, along with a lower recognition threshold for angry faces, despite non-significant differences in recognition accuracy [135]. Similarly, another study reported increased pupil dilation to happy and angry faces under OT, accompanied by enhanced recognition accuracy, particularly among individuals with reduced sensitivity to differences between implicit angry and happy faces [134].

In contrast, the third study (33.3%) found non-significant differences in pupil dilation between the OT and PL conditions [153].

#### Functional magnetic resonance imaging and eye-gazing/pupillometry.

Three studies explored the effects of in-OT administration (two using 24 IU, and one using 8 IU, 48 IU, or 1 IU iv-OT) on brain activation in relation to pupil dilation and/or eye-gazing during the recognition of emotional faces. Two studies employed an explicit task, and one used an implicit task.

In the study examining the relationship between brain activation and pupil dilation, OT (vs. PL) was associated with a general decreased in pupil dilation across all stimuli, including emotional (happy, angry, and neutral faces) and non-emotional (shapes) [146]. Furthermore, under OT, pupil dilation positively correlated with right amygdala activation in response to both emotional and non-emotional stimuli [146].

The remaining two studies examined the relationship between brain activation and eye-gazing patterns. One study (50%) found non-significant OT-related effects on fixation patterns for emotional expressions (angry, fearful, happy, and neutral faces). Nevertheless, OT enhanced activity in several brain areas: the bilateral fusiform gyrus, left superior temporal gyrus, and left amygdala for fearful faces (vs. neutral); the bilateral inferior frontal gyrus for angry faces (vs. neutral); and the left fusiform gyrus and right inferior frontal gyrus for happy faces (vs. neutral) [154].

In contrast, the second study (50%) found increased eye-gazing across emotional expressions (fearful, happy, and neutral faces) under OT, accompanied by enhanced activity in the left amygdala for happy faces and attenuated activity in the left amygdala for fearful faces [67]. Additionally, across emotions, OT was associated with a positive correlation between eye-gazing and right posterior amygdala activation, as well as increased functional connectivity between the right posterior amygdala and the superior colliculus [67].

### Quality assessment

Among the 66 studies evaluated, six were rated as “high” quality, 52 as “medium”, and eight as “low”. A detailed overview of compliance with each criterion, along with the overall quality score and individual criterion scores for each study, is presented in S5 Table.

## Discussion

This scoping review systematically presents findings from 66 studies aimed at characterizing the behavioral, central, and peripheral nervous system correlates of facial emotion recognition, with a focus on psychopathy dimensions and the OT system. We identified a notable gap in research examining the role of OT on psychopathic traits – analyzed as a continuum – in adult populations using a facial emotion recognition paradigm. Consequently, we gathered the evidence into two distinct research subfields: 1) the behavioral, central, and peripheral nervous system correlates of facial emotion recognition within psychopathy dimensions; and 2) the role of OT in these correlates, independent of psychopathy. The following discussion highlights the importance of addressing these research gaps in future studies.

Overall, the results suggest that higher levels of psychopathy affect an individual’s perception, recognition, and response to facial emotions. Individuals with higher psychopathic traits gave greater difficulty identifying and perceiving emotions compared with those with lower traits, whether in forensic or community samples, consistent with existing theoretical models of psychopathy [27,32]. However, this effect may be limited to certain emotions, with the most consistent evidence found for fearful, angry, sad, and disgustful facial expressions.

### Psychopathy, oxytocin, and facial emotion recognition

Most studies (*n* = 13) reporting significant associations between psychopathic traits and emotion recognition accuracy (explicit or implicit) – whether behavioral only or including physiological measures – found poorer performance in recognizing emotional faces, particularly those of negative valence (fearful, disgustful, sad, and angry), although positive valence emotions (happy and surprised) were also affected. These results align with findings from a recent systematic review in children and adolescents with psychopathic traits [155]. However, a few studies (*n* = 2) reported better emotion recognition performance, aligning with the hypothesis that psychopathy may involve higher cognitive empathy alongside lower affective empathy [5,33]. This inconsistency may stem from the lack of differentiation between psychopathy dimensions in the analyses. Notably, the two studies reporting better emotion recognition, which did not consider psychopathy dimensions, were rated as “low” and “medium” in our quality assessment.

In studies that analyzed psychopathy dimensions separately and reported significant results (*n* = 10), both F1- and F2-related traits showed mixed correlations with recognition accuracy for both positive and negative valence emotions. Nevertheless, the most robust association with poorer performance was found for F2-related traits (*n* = 7), consistently with recent studies and a meta-analysis [19,20]. However, this association was inconsistent across valence types (negative, positive, or both). A minority of studies (*n* = 2) found positive associations between F1-related traits and accuracy in recognizing negative emotions, whereas others (*n* = 4) reported poorer performance for these faces. Among studies with non-significant results for accuracy (*n* = 11), four used only behavioral measures (classified as “low”, “medium,” or “high”), and seven also included physiological measures (classified as “medium” or “high”).

These inconsistencies may also be attributed to sample heterogeneity. Some studies recruited community samples, inmates, or psychiatric forensic patients [156], with most samples consisting solely of males. This sex imbalance may result in different patterns of psychopathic traits or sex-related biases in assessment methods, as discussed in the literature [156]. Additionally, the wide range of tasks and types of emotions assessed likely contributed to variability in findings. Furthermore, many studies did not account for cognitive ability, which may explain some variance in emotion recognition performance [113].

Regarding the effects of in-OT on recognition accuracy, OT consistently showed a positive effect on recognizing both positive and negative valence emotions, particularly negative ones, across studies with behavioral-only or those incorporating physiological measures (*n* = 9). These results support the “Social Salience Hypothesis of OT” [26]. Only one study found that OT worsened recognition (by inducing a positive bias for negative valence faces), while a minority (*n* = 6) found non-significant effects on recognition accuracy, response time, or emotional ratings, though these studies were rated as “medium” in quality.

Variability in emotion recognition paradigms, OT dosages, and study sample composition (e.g., only males, only females, or both) may contribute to inconsistencies in findings regarding OT’s effects on behavioral and physiological correlates, as suggested by other studies [157].

### Psychopathy, oxytocin, and central nervous system correlates of facial emotion recognition

Regarding brain function, most fMRI studies (*n* = 6) reported that psychopathic traits are associated with lower activation in brain regions involved in emotion recognition (e.g., fusiform gyrus, inferior frontal gyrus, orbitofrontal cortex, dorsomedial prefrontal cortex, ventromedial prefrontal cortex) during the viewing of emotional faces. Specific findings include amygdala hypoactivation associated with F1-related traits (*n* = 2) and hyperactivation associated with F2-related traits (*n* = 2) when viewing faces of both positive and negative valence, particularly negative ones. These findings align with evidence distinguishing the underlying instrumental (i.e., proactive) and reactive aggression mechanisms [5,27,32,35].

In-OT robustly attenuated amygdala activation in response to emotional faces (*n* = 6) of both positive and negative valence, particularly negative valence. These findings suggest a potential therapeutic approach for reducing the amygdala hyperactivation associated with F2-related reactive aggression [5,35], similar to the normalizing effects of OT in amygdala hyperactivity in SAD [71].

Additionally, two studies combining fMRI and pupillometry reported enhanced amygdala activation for fearful faces and happy faces under OT (vs. PL), one of which used an exclusively female sample. These results corroborate findings that in-OT can normalize amygdala hypoactivation in clinical populations with ASD [71], suggesting that OT may be a potential treatment target for F1-related traits, whether through pharmacological or behavioral interventions. By influencing saliency networks, OT could facilitate social interactions among individuals with psychopathic traits.

Similarly, EEG studies highlight distinct neurocorrelates associated with psychopathy dimensions. Lower N170 amplitudes in response to negative valence emotions were linked to F1-related traits (*n* = 1), while higher amplitudes were associated with F2-related traits for both negative and positive valence emotions (*n* = 1). Only one study, rated as “high” quality, found non-significant associations. Interestingly, N170 is an ERP reflecting early perception of facial features, primarily via the fusiform gyrus and superior temporal sulcus [158,159]. Moreover, OT enhanced N170 amplitudes when participants viewed negative valence emotional faces (*n* = 2), consistent with the “Social Salience Hypothesis of OT” [27,32]. Notably, a recent study found that OT enhanced N170 amplitude during the early perceptual stages of both emotional social (fearful faces) and nonsocial stimuli, indicating a broader role of OT in salience attribution [160].

Regarding eye-gazing, there is again evidence of psychopathy dimension specificity: lower eye-gazing was predominantly associated with F1-related traits (*n* = 3), particularly for negative valence emotions, while higher eye-gazing was suggestively associated with F2-related traits for negative emotions (*n *= 1), reflecting opposite patterns of social salience attribution across F1- and F2-related traits. Only one study found non-significant correlations between eye-gazing and psychopathy dimensions (rated as “medium”), and another reported reduced eye-gazing for both negative and positive valence faces in psychopathic offenders (PCL-R score of at least 25). However, because this latter study did not analyze psychopathy dimensions separately – despite being rated as “high” quality – it remains unclear which dimension primarily contributed to these results. OT also enhanced the salience of social stimuli by facilitating eye-gazing (*n* = 3). The only study reporting non-significant effects on eye-gazing with OT (*n* = 1) was rated as “medium.”

### Psychopathy, oxytocin, and peripheral nervous system correlates of facial emotion recognition

Studies examining peripheral nervous system correlates, such as pupil dilation, showed contradictory results. One study (rated as “high”) found a negative correlation between F1-related traits and pupil dilation in response to negative images and angry faces, and a positive correlation for happy faces. Another study showed a negative association between pupil dilation for fearful, happy, and sad faces and the Meanness subscale, which correlates with both F1- or F2-related traits, rendering it non-specific to either dimension. However, this study was rated as “medium.” The only study reporting no association, despite being classified as “high” quality, was also the only one to use a community sample. In contrast, the other two studies involved forensic samples, which may present different patterns or severity levels of psychopathic traits.

Regarding the effects of in-OT on the autonomic system’s response to emotional faces, results suggest an enhancement of social salience, as evidenced by increased pupil dilation (*n* = 2) for both negative and positive emotions. The only study reporting the opposite effect (a decrease in pupil dilation with OT) (*n* = 1) was rated as “low.”, largely due to methodological limitations, including limited consideration of demographic or neuropsychological variability among participants. Nevertheless, these findings suggest that in-OT may increase emotional arousal and attention to key social cues, which are crucial for emotion recognition and appropriate approach versus avoidance behaviors [39,48,49].

### Summary

This scoping review aimed to explore the effect of OT on behavioral, central, and peripheral correlates of facial emotion recognition in relation to distinct psychopathy dimensions – Interpersonal-affective (F1) and Lifestyle-antisocial (F2) – analyzed as a continuum in adults. Although no studies directly addressed all components of the research question, separate findings on psychopathy and OT suggest distinct and often opposing patterns of impairment within each psychopathy dimension. These correlates appear to be modulated by in-OT administration, frequently in a dimension-specific, counterbalancing manner across F1- and 2- related traits.

For instance, F1-related traits were consistently associated with hypoarousal and reduced sensitivity to emotional stimuli – manifested by hyporeactive amygdala [120,126], decreased attention to the eyes of angry and fearful faces [42,43,123], attenuated pupil dilation [40], and reduced face perception-related N170 response to fearful faces [128]. Such findings help explain core characteristics of F1-related traits, such as blunted affect, low empathy, and lack of guilt, which are often linked to proactive aggression [5,33]. Notably, OT appears to modulate precisely these markers: it enhances attention to the eye region [52,132], pupil dilation [134,135], and N170 amplitude [145] in response to emotional faces. These patterns suggest that OT-based interventions may be promising among individuals with high F1-related traits, aimed at enhancing social salience and well-functioning.

In contrast, F2-related traits are associated with impaired emotion recognition accuracy across emotional valences. There is a concomitant amygdala hyperactivity in response to fearful faces [120,126] and increased attention to the eyes of fearful faces [43]. EEG findings further underscore this domain specificity, with F2-related traits associated with enhanced N170 amplitude for fearful faces [129]. F2-related traits involve a larger environmental (as opposed to genetic) component, often arising from early emotional trauma [161], and are associated with higher levels of anxiety [161] and heightened reactivity to aversive stimuli [5,33]. Unlike F1, these findings aligned with a more impulsive and irresponsible profile, frequently linked to reactive aggression [5,9]. Given OT’s role in improving emotion recognition [131–134] and attenuating amygdala activation – often dampening hyperractivity to social threats [136,143,144,149,150] – in-OT may offer a dimension-specific therapeutic pathway for mitigating social impairments in individuals with high F2-related psychopathic traits.

Considering the study’s initial hypotheses, namely: “1) the OT system may be abnormal in individuals with psychopathic traits, particularly concerning emotion recognition correlates; and 2) OT-based interventions (pharmacological or behavioral) could be promising in addressing these deficits.” The findings of this scoping review offer preliminary yet valuable insights, although no studies meeting the inclusion criteria directly addressed these hypotheses.

Regarding the first hypothesis, evidence from studies including adults with ASPD and residential youths suggests that endogenous OT levels may reflect dimension-specific impairments. Consistently, higher OT levels have been associated with F2-related traits, probably as a result of higher anxiety [86,87]. In contrast, lower OT levels were observed among youths with high CU traits and low emotional neglect – a profile consistent with F1-related traits [89].

In relation to the second hypothesis, several studies support the potential of in-OT in modulating emotion recognition impairments and associated brain activity. Specifically, in-OT reversed amygdala hyperactivation to angry faces among individuals with ASPD (F2-related) [80], while enhanced mid-cingulate cortex hypoactivation in response to fearful faces among those with both ASPD and psychopathy [81]. In-OT also reversed deficits in recognizing fearful faces in both adults with ASPD [83] and youths with conduct disorder, while increasing empathy in youths with high CU traits [84].

More broadly, OT has shown promise in improving social impairments among clinical populations (e.g., schizophrenia, ASD, BPD), though results remain inconsistent [60,71,75]. Variability in OT outcomes appears to be influenced by contextual and individual differences, such as group dynamics (in-group vs. out-group) and specific personality traits [26,64,74].

Studies focusing on reactive aggression paradigms provide complementary and relevant insights into psychopathy. While some findings indicate a reduction in aggression under provocations following OT administration [162–164], others suggest increased aggressive responses under OT [165–169], particularly among individuals with high levels of physical aggressiveness [166] or traits related to interpersonal manipulation and anger [165]. One study involving adults with ASPD found non-significant effects of in-OT on reactive aggression, potentially due to limited statistical power (*n *= 6) [170].

Taken together, these findings suggest that psychopathy dimensions may reflect distinct neurochemical and neurofunctional pathways related to OT, providing preliminary support for the study’s hypotheses. They also underscore the need for dimension-specific approaches in both research and intervention, considering the differential roles OT may play across F1- and F2-related traits. Future studies are crucial to clarify under which conditions OT-based interventions may be beneficial or contraindicated.

### Limitations

This review has some inherent limitations. First, regarding the main objective, we decided to focus on emotion recognition paradigms, given their relevance in both OT and psychopathy literature. Accordingly, we excluded tasks where emotion recognition (implicit or explicit) was not the primary objective (e.g., face memory or facial mimicry), as well as tasks assessing cognitive empathy using only eye stimuli (e.g., the Reading the Mind in the Eyes Test). While this approach ensured consistency in task type and stimuli, it may have led to the omission of potentially relevant findings.

The lack of studies directly addressing the relationship between psychopathy and OT in facial emotion recognition prompted a split-search strategy: one focused on psychopathy and facial emotion recognition; and another on OT and facial emotion recognition. This approach required the indirect integration of findings, which may constrain the strength of the interpretations drawn. Nonetheless, the overlap between psychopathy dimensions and OT-related social salience mechanisms, along with existing evidence from populations with ASPD and CU traits, supports this exploratory synthesis.

We also restricted our inclusion to studies that investigated psychopathy as a continuous variable. This decision excluded studies that only analyzed psychopathy or ASPD as a dichotomous diagnosis, which may have resulted in missing relevant data. However, this criterion aimed to reduce heterogeneity in the operationalization of psychopathy and allowed for a more dimensional understanding of trait-specific effects. Importantly, psychopathic traits can be present across both forensic and community populations, and this variability is critical to understanding associated social and neural processes. Still, some studies not captured by the search or inclusion criteria were discussed to contextualize key findings.

Finally, quality assessment revealed that most included studies were rated as “medium” quality, with few achieving “high” scores. Common methodological limitations included vague hypotheses, insufficient description of recruitment and eligibility criteria, limited consideration of sample heterogeneity, lack of power calculations, and incomplete reporting of statistical procedures or correction for multiple comparisons. We addressed these limitations throughout the discussion to account for potential bias in the interpretation of findings.

## Conclusion and future directions

This scoping review provides preliminary evidence that psychopathic traits are associated with atypical responses to facial emotion recognition tasks and emotion perception more broadly, with distinct behavioral and physiological correlates emerging for each psychopathy dimension. Notably, the Interpersonal-affective (F1) and Lifestyle-antisocial (F2) traits appear to follow diverging – often opposing – patterns across both central and peripheral nervous system correlates. These findings support the need to examine these dimensions separately in relation to emotion recognition and social salience processing.

Although high scores on both F1 and F2 factors are typically required to meet the diagnostic threshold for psychopathy [9], studying these traits dimensionally offers valuable insights into their neurobiological underpinnings. We recommend further dimension-wise studies to identify predominant brain activity and behavioral patterns, as some studies are beginning to explore.

With regard to OT, current findings suggest its potential as a pharmacological target for improving social impairments in individuals with psychopathic traits. Future studies should continue to explore OT’s role in psychopathy through both pharmacological and endogenous approaches, with a particular emphasis on factorial models of psychopathy. This includes rigorous assessment of both F1- and F2- related traits and facets, as well as considering trait-context interactions. The development of OT-based biomarkers may also support the differential diagnosis of psychopathy dimensions and inform earlier prevention strategies and more tailored interventions.

Nonetheless, whether dysregulation of the OT system can fully account for the neurochemical profiles of psychopathy dimensions – or psychopathy as a whole – remains an open question. Longitudinal and intervention-based studies using multimodal methodologies (e.g., behavioral tasks, neuroimaging, and psychophysiological measures) are especially needed to assess causality and clarify OT’s therapeutic potential in psychopathy.

## Supporting information

S1 File Additional information about psychopathy’s instruments. (PDF)

S2 File Preferred Reporting Items for Systematic reviews and Meta-Analyses extension for Scoping Reviews (PRISMA-ScR) Checklist. (PDF)

S1 Table Search queries per theme section of the scoping review. It was selected the option “All fields” on the search builder of the databases.
(PDF)

S2 Table A 12-criteria score list to appraise the quality of the studies included in this review and the percentage of studies complying the highest score (from 0 to 3) within each criterion. (PDF)

S3 Table Studies on the association of psychopathy and facial emotion recognition performance and functional central and peripheral nervous system correlates. PCL-R = Psychopathy Checklist – Revised; PCL:SV = Psychopathy Checklist: Screening Version; PPI = Psychopathic Personality Inventory; PPI-R = Psychopathic Personality Inventory – Revised TriPM = Triarchic Psychopathy Measure; LSRP = Levenson’s Self-Report Psychopathy Scale; SRP = Self-Report Psychopathy Scale; SRP-III = Self-Report Psychopathy Scale – Third Version; SRP-SF = Self-Report Psychopathy Scale – Short Form; F1 = PCL-R’s Factor 1; F2 = PCL-R’s Factor 2; FG = fusiform gyrus; IFG = inferior frontal gyrus; OFC = orbitofrontal cortex; PFC = prefrontal cortex; mPFC = medial PFC; dmPFC = dorsomedial PFC; vmPFC = ventromedial PFC; SMA = supplementary motor area; INS = insula; aINS = anterior INS; STS = superior temporal sulcus; pSTS = posterior STS; ACC = anterior cingulate cortex; AMY = amygdala; GM = grey matter; ERN = error-related negativity; Pe = error positivity; LPP = late-positive potential; fMRI = functional magnetic imaging; EEG = electroencephalography; ERP = event-related potentials; M age = mean age; ↓ = lower; ↑ = higher; ↓ a = lower activation; ↑ a higher activation; ↑ v = higher volume; n.s. = non-significant; M = males; F = females; HC = healthy controls; R = right; L = left.
(PDF)

S4 Table Studies on the effect of oxytocin intranasal administration or endogenous level on facial emotion recognition performance and functional central and peripheral nervous system correlates. OT = oxytocin; in-OT = intranasal OT; IU = international units; iv-OT = intravenous OT; PL = placebo; AMY = amygdala; ACC = anterior cingulate cortex; FG = fusiform gyrus; STG = superior temporal gyrus; IFG = inferior frontal gyrus; EPN = earlier posterior negativity; INS = insula; OFC = orbitofrontal cortex; M age = mean age; ↓ = lower; ↑ = higher; ↓ a = lower activation; ↑ a higher activation; ↑ c = higher connectivity; ↓ c = lower connectivity; M = males; F = females; R = right; L = left.
(PDF)

S5 Table Quality scores for each study included in this review, assessed from 0 to 3 across the preestablished 12-criteria list. Q1. Were the hypothesis and objectives of the study clearly described? Q2. Have the authors used methods that can test their hypothesis? Q3. Were the recruitment procedure and eligibility criteria for the participants clearly described (e.g., ethnicity, age range, gender, neuropsychiatric and other medical conditions, handedness)? Q4. Was there an effort to account for demographic or neuropsychological variability amongst participants (e.g., age, gender, handedness, IQ, level of education, smoking, alcohol/drug use and, for cases, duration of illness and medication status)? Q5. Did the study include an a priori power calculation to define sample sizes? Q6. Was a valid cognitive task assigned to elicit the desired effects in the participants and it was clearly described? Q7. Was the assessment of psychopathy performed with valid measurement tools and clearly described, when applied? Q8. Were aspects related to oxytocin administration or measurement adequate and clearly described, when performed (e.g., dose, administration method, time of administration, laboratory technique for oxytocin quantification)? Q9. In studies with central and/or peripheral nervous system activity assessment, the technique, respective parameters and preprocessing were outlined? Q10. Was the statistical methodology clearly explained? Q11. Was a consensual statistical significance threshold set (i.e.,., p < 0.05 after correction for multiple comparisons)? Q12. Were effect sizes reported for significant results (e.g., Cohen’s d, Pearson’s correlation, odds ratio or risk ratios)? The general quality of the study was obtained by dividing the sum of the 12 items’ scores by the maximum sum applicable to each study. N/A = Not applicable.
(PDF)
