# Supplementary material for: Psychophysiology of facial emotion recognition in psychopathy dimensions and oxytocin’s role: A scoping review
Source: PLoS One. 2025 Jul 30;20(7):e0327764. doi: 10.1371/journal.pone.0327764 (PMC12310049; doi:10.1371/journal.pone.0327764)
Supplement: S1 File — (PDF) [file pone.0327764.s001.pdf]

S1 File. Additional information about psychopathy's instruments

The original PCL-R has a short 12-item version with the same facets (Hart et al., 1999) called the PCL:SV which is adequate for use outside forensic settings [1,2] such as scientific research [3].

The SRP [4] also derives from the original PCL-R. It is a self-report psychopathy scale, typically applied as a questionnaire, with a five-point Likert scale, assessing the same four facets as above [4]. Many scientific studies from various countries and various types of forensic samples conclude that the PCL-R scores are highly reliable when used by trained and experienced raters. Additionally, the scale demonstrates high internal consistency and inter-item correlations, making it a valid instrument for psychopathy assessment (Hare et al., 2000).

The TriPM [6] is a self-report scale composed of four-point Likert scale items, each under one of three subscales: Meanness, Boldness and Disinhibition. They are based on the triarchic conceptualization of psychopathy by Patrick, Fowles, and Krueger [7], which is a model aiming to capture specific psychopathy elements that the PCL-R does not, such as Boldness [8]. TriPM's scales have been demonstrated to have weak to moderate correlations with PCL-R total scores, such as: Boldness with Interpersonal, Disinhibition with Lifestyle, Meanness with Lifestyle, and Interpersonal and Antisocial [8–10].

The PPI [11] is a self-report psychopathy instrument consisting of two factors (PPI-I and PPI-II) and eight subscales, which include Fearlessness, Social Potency and Stress Immunity (which fall under PPI-I), Carefree Nonplanfulness, Egocentricity, Machiavellianism, Blame Externalization and Impulsive Nonconformity (which load on PPI-II), and Coldheartedness (which falls under neither factor). The factor PPI-I demonstrates

moderate but significant correlations with the PCL-R's F1, and the PPI-II with the PCL-R's F2 [12].

Lastly, the LSRP [13] is a self-report instrument with a very easy reading level, making it more accessible to a wider population. It consists of items endorsing a four-point scale of agreement with each specific statement. It consists of two subscales: Primary Psychopathy and Secondary Psychopathy, which are similar, in conception, to the PCL-R's F1 and F2, respectively factors – both scales showing a moderate yet significant correlation in scores [12].

References

1. Coid J, Yang M, Ullrich S, Roberts A, Hare RD. Prevalence and correlates of psychopathic traits in the household population of Great Britain. Int J Law Psychiatry. 2009;32: 65–73. doi:10.1016/j.ijlp.2009.01.002
2. Neumann CS, Hare RD. Psychopathic Traits in a Large Community Sample: Links to Violence, Alcohol Use, and Intelligence. J Consult Clin Psychol. 2008;76: 893–899. doi:10.1037/0022-006X.76.5.893
3. Cooke DJ, Michie C, Hart SD, Hare RD. Evaluating the Screening Version of the Hare Psychopathy Checklist—Revised (PCL:SV): An item response theory analysis. Psychol Assess. 1999;11: 3–13. doi:10.1037/1040-3590.11.1.3
4. Hare R, Neumann C. Psychopathy as a clinical and empirical construct. Annu Rev Clin Psychol. 2008;4: 217–246. doi:10.1146/annurev.clinpsy.3.022806.091452
5. Hare RD, Clark D, Grann M, Thornton D. Psychopathy and the predictive validity of the PCL-R: an international perspective. Behavioral sciences & the law. 2000;18: 623–645. doi:10.1002/1099-0798(200010)18:5<623::aid-bsl409>3.0.co;2-w

6. Patrick CJ. Operationalazing the Triarchic Conceptualization of Psychopathy: Preliminary Description of Brief Scales for Assessment of Boldness, Meanness, and Disinhibition. Unpublished test manual, Florida State University, Tallahassee, Florida; 2010.
7. Patrick CJ, Fowles DC, Krueger RF. Triarchic conceptualization of psychopathy: developmental origins of disinhibition, boldness, and meanness. Dev Psychopathol. 2009;21: 913–938. doi:10.1017/S0954579409000492
8. Evans L, Tully RJ. The Triarchic Psychopathy Measure (TriPM): Alternative to the PCL-R? Aggress Violent Behav. 2016;27: 79–86. doi:10.1016/j.avb.2016.03.004
9. Sleep CE, Weiss B, Lynam DR, Miller JD. An examination of the Triarchic Model of psychopathy's nomological network: A meta-analytic review. Clin Psychol Rev. 2019;71: 1–26. doi:10.1016/j.cpr.2019.04.005
10. Wall TD, Wygant DB, Sellbom M. Boldness explains a key difference between psychopathy and antisocial personality disorder. Psychiatry, Psychology and Law. 2015;22: 94–105. doi:10.1080/13218719.2014.919627
11. Benning SD, Patrick CJ, Hicks BM, Blonigen DM, Krueger RF. Factor Structure of the Psychopathic Personality Inventory: Validity and Implications for Clinical Assessment. Psychol Assess. 2003;15: 340–350. doi:10.1037/1040-3590.15.3.340
12. Poythress NG, Lilienfeld SO, Skeem JL, Douglas KS, Edens JF, Epstein M, et al. Using the PCL-R to help estimate the validity of two self-report measures of psychopathy with offenders. Assessment. 2010;17: 206–219. doi:10.1177/1073191109351715
13. Levenson MR, Kiehl KA, Fitzpatrick CM. Assessing Psychopathic Attributes in a Noninstitutionalized Population. J Pers Soc Psychol. 1995;68: 151–158. doi:10.1037/0022-3514.68.1.151
