# Supplementary material for: Psychophysiology of facial emotion recognition in psychopathy dimensions and oxytocin’s role: A scoping review
Source: PLoS One. 2025 Jul 30;20(7):e0327764. doi: 10.1371/journal.pone.0327764 (PMC12310049; doi:10.1371/journal.pone.0327764)
Supplement: S1 Table — It was selected the option “All fields” on the search builder of the databases. (PDF) [file pone.0327764.s003.pdf]

S1 Table. Search queries per theme section of the scoping review

**Psychopathy, oxytocin and emotion recognition**
(psychopathy OR psychopathic) AND oxytocin AND (facial OR face) AND (emotion recognition OR passive viewing) AND adult
**Psychopathy and emotion recognition**
(psychopathy OR psychopathic) AND (facial OR face) AND (emotion recognition OR passive viewing) AND adult
(psychopathy OR psychopathic) AND (facial OR face) AND (emotion recognition OR passive viewing) AND adult AND (fMRI OR “functional magnetic resonance” OR neuroimaging)
(psychopathy OR psychopathic) AND (facial OR face) AND (emotion recognition OR passive viewing) AND adult AND (EEG OR electroencephalography OR ERP OR evoked potentials)
(psychopathy OR psychopathic) AND (facial OR face) AND (emotion recognition OR passive viewing) AND adult AND (“eye-tracking” OR eye-gaze OR saccade)
(psychopathy OR psychopathic) AND (facial OR face) AND (emotion recognition OR passive viewing) AND adult AND pupil*
**Oxytocin and emotion recognition**
oxytocin AND (facial OR face) AND (emotion recognition OR passive viewing) AND adult
oxytocin AND (facial OR face) AND (emotion recognition OR passive viewing) AND adult AND (fMRI OR “functional magnetic resonance” OR neuroimaging)
oxytocin AND (facial OR face) AND (emotion recognition OR passive viewing) AND adult AND (EEG OR electroencephalography OR ERP OR evoked potentials)
oxytocin AND (facial OR face) AND (emotion recognition OR passive viewing) AND adult AND (“eye-tracking” OR eye-gaze OR saccade)
oxytocin AND (facial OR face) AND (emotion recognition OR passive viewing) AND adult AND pupil*

Note. It was selected the option “All fields” on the search builder of the databases.
