# Supplementary material for: Psychophysiology of facial emotion recognition in psychopathy dimensions and oxytocin’s role: A scoping review
Source: PLoS One. 2025 Jul 30;20(7):e0327764. doi: 10.1371/journal.pone.0327764 (PMC12310049; doi:10.1371/journal.pone.0327764)
Supplement: S3 Table — PCL-R = Psychopathy Checklist – Revised; PCL:SV = Psychopathy Checklist: Screening Version; PPI = Psychopathic Personality Inventory; PPI-R = Psychopathic Personality Inventory – Revised TriPM = Triarchic Psychopathy Measure; LSRP = Levenson’s Self-Report Psychopathy Scale; SRP = Self-Report Psychopathy Scale; SRP-III = Self-Report Psychopathy Scale – Third Version; SRP-SF = Self-Report Psychopathy Scale – Short Form; F1 = PCL-R’s Factor 1; F2 = PCL-R’s Factor 2; FG = fusiform gyrus; IFG = inferior frontal gyrus; OFC = orbitofrontal cortex; PFC = prefrontal cortex; mPFC = medial PFC; dmPFC = dorsomedial PFC; vmPFC = ventromedial PFC; SMA = supplementary motor area; INS = insula; aINS = anterior INS; STS = superior temporal sulcus; pSTS = posterior STS; ACC = anterior cingulate cortex; AMY = amygdala; GM = grey matter; ERN = error-related negativity; Pe = error positivity; LPP = late-positive potential; fMRI = functional magnetic imaging; EEG = electroencephalography; ERP = event-related potentials; M age = mean age; ↓ = lower; ↑ = higher; ↓ a = lower activation; ↑ a higher activation; ↑ v = higher volume; n.s. = non-significant; M = males; F = females; HC = healthy controls; R = right; L = left. (PDF) [file pone.0327764.s005.pdf]

S3 Table. Studies on the association of psychopathy and facial emotion recognition performance and functional central and peripheral nervous system correlates

Study	Sample	Psychopathy measure/scores	Task/Emotional stimuli	Association with psychopathy
**Behavior (accuracy)**				
Beussink et al., 2020	N total = 145 M offenders; age = 26.26±6.86	PCL-R total and subscales (whole sample)	Explicit recognition of emotions: Matching of emotions (angry, disgustful, sad, surprised, fearful and happy faces)	↑ accuracy for fearful faces (Affective facet)<br>↓ accuracy for happy faces (Lifestyle facet)
Brislin & Patrick, 2019	N total = 127; M (n = 62); F (n = 65); age = 19.5±3.7	TriPM total and subscales (whole sample)	Explicit recognition of emotions (angry, disgustful, fearful, happy, sad and surprised faces, with different intensities)	↓ accuracy for middle-intensity fearful faces (Meanness subscale)<br>↓ accuracy for low-intensity disgustful faces (Disinhibition subscale)
Contreras-rodríguez et al., 2014	N=44 M; criminal offenders (n=22; age=39.8±9.2); healthy nonoffenders (n=22; age=40.6±9.5)	PCL-R total and subscales (whole sample)	Implicit emotional face-matching task (happy, fearful and angry faces)	Accuracy: n.s.
Dargis et al., 2018	N = 198 M inmates; age = 18-55	PCL-R total and subscales (whole sample)	Explicit recognition of emotions (happy, sad, angry, fearful, disgustful, and neutral faces) and viewing of emotions (happy, angry, fearful, and neutral faces)	↓ accuracy for fearful faces (Affective facet)<br>↑ accuracy for fearful faces (Lifestyle facet)
Deeley et al., 2006	N total = 15 M; offenders (n = 6; age = 36.0±9.0); HC (n = 9, age = 27.0±5.0)	PCL-R total (offenders)	Implicit emotion recognition task (fearful, happy and neutral faces)	Accuracy: n.s.

Study	Sample	Psychopathy measure/scores	Task/Emotional stimuli	Association with psychopathy
Dolan & Fullam, 2006	N total = 98 M; offenders (n = 49, age = 35.2±10.3); HC (n = 49, ag e =35.3±9.1)	PCL:SV total and subscales (offenders)	Explicit recognition of emotions (morphed angry, disgustful, fearful, happy, sad and surprised faces, with different intensities)	↓ accuracy for sad faces (total score)<br>↓ accuracy for happy faces (Antisocial facet)<br>Response time: n.s
Dolan & Fullam, 2004	N total = 109 M; antisocial inmates (n = 89; age = 36.7±9.9); HC (n = 20; age = 33.6±4.9)	PCL:SV total (anti-social inmates)	Explicit recognition of emotions (happy, sad, angry, fearful, surprised, disgustful and distressed faces, with covered or uncovered eyes)	↑ accuracy for all emotion expressions (antisocial inmates)
Eisenbarth et al., 2008	N total = 44 F; forensic patients (n = 28) + community people (n = 16); age = 41.7±11.5	PCL-R total (forensic patients)	Explicit recognition of emotions (anxious, angry, disgustful, happy, neutral, sad and surprised)	↓ accuracy for short presentation of sad faces
Faith et al., 2023	N total = 139 M; inmates; age = 26.3±6.9	PCL-R total and subscales (whole sample)	Explicit recognition of emotions (morphed happy, sad, angry, fearful, surprised and disgusted faces)	↑ response time for angry faces (Antisocial facet)
Faith et al., 2022	N total = 288 M; inmates; age = 26.7±6.8	PCL-R total (whole sample)	Explicit recognition of emotions (morphed happy, sad, angry, fearful, surprised and disgusted faces)	↓ accuracy for fearful faces
Gehrer et al., 2019	N total = 36 M; psychopathic (n = 19, age = 40.3±11.1); non-psychopathic offenders (n = 17, age = 37.4±9.0)	PCL-R total (whole sample)	Explicit recognition of emotions & gender (angry, disgustful, happy, fearful, sad, surprised and neutral faces)	Accuracy: n.s.
Gillespie et al., 2019	N total = 73 M violent offenders; age = 38.7±11.7	TriPM total and subscales (whole sample)	Explicit recognition of emotions (morphed neutral, angry, disgustful, fearful, happy, sad, and surprised faces with different levels of intensity)	↓ accuracy for fearful faces<br>↑ accuracy for sad faces

Study	Sample	Psychopathy measure/scores	Task/Emotional stimuli	Association with psychopathy
Gillespie et al., 2017	N total = 55 M; violent offenders (n=30; age = 35.1±11.8; community people (n = 25; age = 37.9±18.3	TriPM subscales (whole sample)	Explicit recognition of emotions (morphed angry, disgustful, fearful, happy, sad and surprised faces, with different intensities)	Accuracy: n.s.
Gillespie et al., 2015	N = 38 community M; age = 23.2±4.9	LSRP subscales (whole sample)	Explicit recognition of emotions (morphed angry, disgustful, fearful, happy, sad and surprised faces, with different intensities)	Accuracy: n.s.
Glass & Newman, 2006	N total = 111 M; psychopathic inmates (n = 50, age = 32.58±7.08); non-psychopathic inmates (n = 61, age = 32.0±7.0)	PCL-R total score (whole sample)	Explicit recognition of emotions (angry, fearful, happy and sad faces): Identify condition & Locate condition (congruent with attentional set)	↑ accuracy for fearful faces (Identify condition)
Gordon et al., 2004	N total = 20 community M; age = 23.5±4.1	PPI total and subscales (whole sample)	Explicit recognition of emotions (angry, fearful, sad, and joyful faces)<br>Emotion recognition condition and identity condition (which emotion is vs. who the person is)	Accuracy: n.s.<br>Response time: n.s.
Hansen et al., 2008	N total = 43 M offenders; age = 31.6	PCL-R total and subscales (whole sample)	Explicit recognition of emotions (sad, fearful, disgustful, angry, surprised, happy, and neutral)	↑ accuracy for disgustful faces (Lifestyle and Antisocial facets)<br>↓ accuracy for neutral faces (Affective facet)<br>↓ accuracy for female disgustful faces (Interpersonal facet)

Study	Sample	Psychopathy measure/scores	Task/Emotional stimuli	Association with psychopathy
Igoumenou et al., 2017	N total = 1353 M ex-inmates; age = 29.9±0.4	PCL-R total and subscales (whole sample)	Explicit recognition of emotions (morphed happy, surprised, sad, fearful, angry and disgustful faces, with different intensities)	↑ accuracy for angry faces (Interpersonal facet)<br>↓ accuracy for fearful and disgustful faces (Affective facet)<br>↓ accuracy for surprised faces (Lifestyle facet)<br>↓ accuracy for fearful, disgustful and surprised faces (Antisocial facets)
Jusyte & Schönenberg, 2017	N total = 69 M; violent offenders (n = 34; age = 37.8±10.84); HC (n = 35; age = 30.5±11.8)	SRP-III total and subscales (whole sample)	Implicit recognition of emotions<br>Experiment 1: Emotion sensitivity (angry, happy, fearful and neutral faces, with different intensities);<br>Experiment 2: Ambiguous expressions task (blended happy-fearful, happy-angry and fearful-angry faces)	Experiment 1: n.s.<br>Experiment 2: ↓ accuracy for ambiguous fearful faces (violent offenders' total score, Callous Affect and Antisocial Behavior subscales)
Khvatskaya & Lenzenweger, 2016	N total = 47 community; M (n = 16); F (n = 31); age = 18.9±1.6	PPI-R total (whole sample)	Explicit recognition of emotions (angry, contempted, disgustful, fearful, joyful, sad, surprised, or neutral faces)	n.s.
Kosson et al., 2002	N total = 67 M; psychopath inmates (n = 34, age = 27.0±2.8); non-psychopath inmates (n = 33, age=27.0±6.5)	PCL-R total (whole sample)	Explicit recognition of emotions (happy, sad, angry, fearful and disgustful)	↓ recognition accuracy for disgustful faces
Kranefeld & Blickle, 2022	N total = 477 community; M (n = 305); F (n = 272); age = 40.3±12.3	TriPM, PPI-R and LSRP total and	Implicit emotional face-matching task	Accuracy: n.s.

Study	Sample	Psychopathy measure/scores	Task/Emotional stimuli	Association with psychopathy
		subscales (whole sample)		
Kuin et al., 2017	N total = 117 M; violent offenders (n=71; age = 36.6±11.8); nonviolent offenders (n = 14; age = 37.4±10.4); HC (n = 32; age = 41.8±11.2)	PPI-R total (violent offenders and nonviolent offenders)	Explicit recognition of emotions (morphed sequences from unambiguous happy to unambiguous angry faces)	n.s.
Künecke et al., 2018	N total = 266 M; inmates (n = 179) and HC (n = 87); age = 35.9±11.0	PCL:SV total (whole sample)	Explicit recognition of emotions (morphed dynamic angry, happy, sad and neutral faces)	↑ response time for neutral faces
Mier et al., 2014	N total = 29 M; inmates (n = 11, age = 44.6±9.0); HC (n = 18, age = 44.0±10.4)	PCL-R total (inmates); PPI-R total (HC)	Explicit recognition of emotions (happy, angry, fearful and neutral faces): Affective Theory of Mind (ToM), Emotion recognition & Neutral face conditions	Accuracy: n.s.<br>Response time: n.s
Mowle et al., 2019	N total = 110 community; M (n = 36); F (n = 74); age 18-19	TriPM total and subscales (whole sample)	Explicit recognition of emotions (fearful, happy, sad, angry, surprised, calm, neutral, and disgustful faces)	↓ accuracy for fearful faces (Meanness subscale in high traits)
Munro et al., 2007	N total = 30 M; violent offenders (n = 15, age = 45.9±3.5); HC (n = 15, age = 46.6±1.8)	PCL-R total (violent offenders)	Explicit emotion recognition: Face flanker task (angry and fearful faces) and letter flanker task	Response time: n.s.<br>↓ fearful-face flanker accuracy (in violent offenders)

Study	Sample	Psychopathy measure/scores	Task/Emotional stimuli	Association with psychopathy
Olderbak et al., 2018	N total = 339 M; forensic patients + inmates (n = 226) HC group (n = 113); age = 18-65	PCL:SV total (whole sample)	Explicit recognition of emotions (angry, disgustful, fearful, happy, sad and surprised faces):<br>Task 1: Different intensity from upright and inverted dynamic faces;<br>Task 2: Composite faces;<br>Task 3: Facially expressed emotions	Task 1: ↓ accuracy for upright inverted disgustful, fearful, happy and surprised faces (forensic)<br>Task 2: ↓ accuracy for happy expressions in composite (forensic)<br>Task 3: ↓ accuracy for angry faces in inmates and happy (community)
Oliver et al., 2015	N total = 40 community; M (n = 16); F (n = 24); age = 21.6±3.7	PPI-R total and subscales (whole sample)	Implicit recognition of emotions: Continuous flash suppression/presentation (disgustful, fearful and neutral faces); Objective index (emotion localization) & Subjective index (confidence rating)	↓ accuracy for fearful faces (Objective index, Coldheartedness subscale)
Pera-Guardiola et al., 2016	N total = 39 M; criminal offenders (n = 19; age = 39.2±8.9); healthy non-offenders (n = 20; age = 40.6±9.9)	PCL-R total and subscales (whole sample)	Explicit facial emotion expression recognition task (gradually morphed happy, surprised, fearful, sad, disgustful and angry faces)	↓ accuracy for sad, happy and fearful faces<br>↑ accuracy for sad faces and fearful faces (Antisocial facet)
Pham & Philippot, 2010	N total = 68 M; psychopathic inmates (n = 20, age = 34.0±10.1); non-psychopathic inmates (n = 23, age = 34.6±8.8); HC (n = 25, age = 35.4±7.9)	PCL-R total and subscales (inmates)	Explicit recognition of emotions (morphed happy, angry, sad, fearful, or disgustful faces, varied in intensity)	↓ accuracy for happy, angry and disgustful faces

Study	Sample	Psychopathy measure/scores	Task/Emotional stimuli	Association with psychopathy
Philipp-Wiegmann et al., 2017	N = 70 M; reactive violent offenders (n = 17, age = 34.9±14.6); proactive violent offenders (n = 24, age = 34.0±14.5); community people (n = 29, age = 31.6±12.3)	PCL:SV total (whole sample)	Explicit recognition of emotions (angry, fearful, surprised, sad, happy, and disgustful)	↓ accuracy overall (both violent offenders' groups)
Schönenberg & Jusyte, 2014	N total = 110 M; violent offenders (n = 55; age = 33.4±9.8); HC (n = 55; age = 30.4±10.2)	PPI-R total and subscales (violent offenders)	Implicit recognition of emotions: Ambivalence task (morphed sequences happy–fearful, happy–angry and fearful–angry faces)	n.s.
**fMRI**				
Carré et al., 2013	N total = 200; M (n = 83); F (n = 117); community; age = 19.7±1.3	SRP-SF subscales (whole sample)	Implicit emotion perceptual face-matching task (fearful, angry, surprised, and neutral faces)	↓ R dorsal AMY to fearful faces (Interpersonal facet)<br>↑ R dorsal AMY to angry faces (Lifestyle facet)
Contreras-rodríguez et al., 2014	N = 44 M; criminal offenders (n = 22; age = 39.8±9.2); healthy nonoffenders (n = 22; age = 40.6±9.5)	PCL-R total and subscales (whole sample)	Implicit emotional face-matching task (happy, fearful and angry faces)	↑ frontal cortex for all emotions (F1)<br>↓ frontoparietal cortex, visual areas, and diencephalic–mesencephalic structures for all emotions (F2)<br>Connectivity: n.s. (total score, F1 and F2)
Decety et al., 2014	N total = 80 M inmates; age = 18-50	PCL-R total and subscales (whole sample)	Implicit recognition of dynamic emotions (fearful, sad, happy, and painful faces)	F1 and 2: ↓ bilateral FG, R IFG, R OFC,R dmPFC, L inferior temporal pole, and bilateral middle frontal gyrus for happy; ↓ bilateral middle occipital

Study	Sample	Psychopathy measure/scores	Task/Emotional stimuli	Association with psychopathy
				gyrus ,R IFG, and R supramarginal gyrus for fearful faces; ↓ L pSTS, R IFG, bilateral dmPFC, and R SMA for sad; ↓ middle cingulate cortex, IFG, dmPFC and L angular gyrus to painful faces; ↑ aINS for painful faces<br><br>Factor 1:↓right middle occipital gyrus, bilateral IFG,R vmPFC, L OFC and R inferior temporal pole for happy faces; ↓ L insula, R vmPFC, OFC and R SMA for fearful faces; ↓ L IFG and R middle frontal gyrus for sad faces; ↓ R angular gyrus and L pSTS for painful faces; ↑ L postcentral gyrus and R precentral gyrus for painful faces<br><br>Factor 2: ↓ right supramarginal gyrus and R SMA for happy faces; ↓R INS, L IFG, L middle frontal gyrus, and L SMA for fearful faces; ↓ L FG, left IFG and L inferior temporal pole for sad; ↓R STS, dorsal ACC, and striatum for painful faces;

Study	Sample	Psychopathy measure/scores	Task/Emotional stimuli	Association with psychopathy
				Total high vs. low: ↓ bilateral FG, dmPFC, inferior temporal pole, and R middle frontal gyrus for fearful faces<br><br>Factor 1 high vs. low: ↑R INS for fearful faces; ↑left aINS and L middle cingulate gyrus to sad faces
Deeley et al., 2006	N total = 15 M; offenders (n = 6; age = 36.0±9.0) HC (n = 9, age = 27.0±5.0)	PCL-R total (offenders)	Implicit emotion recognition task (fearful, happy and neutral faces)	↓ bilateral cerebellum and FG, L postcentral gyrus for fearful faces (vs. HC)<br>↑ R INS and precuneus, ↓ cerebellum and FG for fearful faces (vs. neutral)<br>↓ R FG, L lingual gyrus, cerebellum and precentral gyrus for happy faces (vs. HC)<br>↑ R cerebellum, bilateral FG and middle occipital gyrus, R precuneus, anterior cingulate gyrus, medial frontal gyrus, L superior parietal lobule and precuneus for fearful faces (vs. neutral)
Gordon et al., 2004	N total = 20 community M; age = 23.5±4.1	PPI total and subscales (whole sample)	Explicit affect recognition task (angry, fearful, sad, and joyful faces)<br>Emotion recognition condition and identity condition (which emotion is vs. who the person is)	↓ R inferior frontal córtex, R AMY and mPFC ↑ visual cortex (Emotional Interpersonal subscale)<br>↑ R AMY in emotion recognition condition (Social Deviance subscale)

Study	Sample	Psychopathy measure/scores	Task/Emotional stimuli	Association with psychopathy
				↓ mPFC in emotion recognition condition (total score)
Mier et al., 2014	N total = 29 M; inmates (n = 11, age = 44.6±9.0); HC (n = 18, age = 44.0±10.4)	PCL-R total (inmates); PPI-R total (HC)	Explicit recognition of emotions (happy, angry, fearful and neutral faces): Affective Theory of Mind (ToM), Emotion recognition & Neutral face conditions	↓ R FG for all faces<br>↓ R FG and L AMY (affective ToM task)
Pera-Guardiola et al., 2016	N total = 39 M; criminal offenders (n = 19; age = 39.2±8.9); healthy non-offenders (n = 20; age = 40.6±9.9)	PCL-R total and subscales (whole sample)	Explicit facial emotion expression recognition task (gradually morphed happy, surprised, fearful, sad, disgustful and angry faces)	↑ volume of dmPFC for better sad faces recognition; and middle ACC, aINS, IFG, OFC and anterior cerebellum for better happy faces recognition; and somatosensory cortex for better fearful faces recognition<br>↑volume of dmPFC for better recognition of all faces (Lifestyle facet)
**EEG**				
Almeida et al., 2014	N total = 54 community M; age = 23.2±3.8	PPI-R total and subscales (whole sample)	Explicit recognition of emotions (angry, fearful, disgustful, happy, neutral and calm faces, varying spatial frequency content)	↓ N170 amplitude for facial expressions (Fearless dominance subscale)<br>↑ N170 amplitude for fearful and happy faces (Coldheartedness subscale)
Brislin & Patrick, 2019	N total = 127; M (n = 62); F (n = 65); age = 19.5±3.7	TriPM total and subscales (whole sample)	Explicit recognition of emotions (angry, disgustful, fearful, happy, sad and surprised faces, with different intensities)	↓ L and R N170 amplitude for fearful faces; ↓ P200 amplitude for fearful faces; and ↓ LPP amplitude to fearful and sad faces (Meanness subscale)

Study	Sample	Psychopathy measure/scores	Task/Emotional stimuli	Association with psychopathy
				↑ L N170 amplitude for fearful faces (Disinhibition subscale)
Eisenbarth et al., 2013	N total = 23 F forensic patients; M = 37.0±8.8	PCL-R total and subscales (whole sample)	Implicit recognition of emotions (fearful, angry, and happy faces); Viewing of emotions (positive, negative and neutral)	N170, P3 and LPP amplitudes: n.s.<br>↓ N2 amplitude for angry and fearful faces (F2)
Munro et al., 2007	N total = 30 M; violent offenders (n = 15, age = 45.9±3.5); HC (n = 15, age = 46.6±1.8)	PCL-R total (violent offenders)	Explicit emotion recognition: Face flanker task (angry and fearful faces) and letter flanker task	↓ ERN negative amplitude during face flanker task (in violent offenders)
**Eye-gazing**				
Dargis et al., 2018	N = 198 M inmates; age = 18-55	PCL-R total and subscales (whole sample)	Explicit recognition of emotions (happy, sad, angry, fearful, disgustful, and neutral faces) and viewing of emotions (happy, angry, fearful, and neutral faces)	↓ fixations on eyes of fearful faces (Interpersonal facet)<br>↑ fixations on eyes of fearful faces (Antisocial facet)<br>↓ fixations on eyes of fearful faces during free viewing (total score)
Gehrer et al., 2019	N total = 36 M; psychopathic (n = 19, age = 40.3±11.1); non-psychopathic offenders (n = 17, age = 37.4±9.0)	PCL-R total (whole sample)	Explicit recognition of emotions & gender (angry, disgustful, happy, fearful, sad, surprised and neutral faces)	↓ dwell time on eyes overall (in psychopathic offenders)
Gillespie et al., 2017	N total = 55 M; violent offenders (n = 30; age =	TriPM subscales (whole sample)	Explicit recognition of emotions (morphed angry, disgustful, fearful,	↓ dwell time and fixations on the eyes overall (Boldness subscale)

Study	Sample	Psychopathy measure/scores	Task/Emotional stimuli	Association with psychopathy
	35.1±11.8; community people (n = 25; age = 37.9±18.3		happy, sad and surprised faces, with different intensities)	
Gillespie et al., 2015	N = 38 community M; age = 23.2±4.9	LSRP subscales (whole sample)	Explicit recognition of emotions (morphed angry, disgustful, fearful, happy, sad and surprised faces, with different intensities)	↓ dwell time and fixations on the eyes of angry and fearful faces (Primary psychopathy scale)
Mowle et al., 2019	N total = 110 community; M (n = 36); F (n = 74); age 18-19	TriPM total and subscales (whole sample)	Explicit recognition of emotions (fearful, happy, sad, angry, surprised, calm, neutral, and disgustful faces)	Fixation on facial features: n.s.
**Pupillometry**				
Burley et al., 2017	N total = 102 community M (n = 50); F (n = 52); age = 21.1±3.6	TriPM total and subscales (whole sample)	Implicit recognition of emotions (unpleasant, pleasant and neutral); Static & dynamic expressions viewing (fearful, happy, neutral, disgustful, angry and sad faces)	n.s.
Burley et al., 2019	N total = 82 M forensic psychiatric inpatients; age = 38.6±12.8	PCL-R total and subscales (whole sample)	Implicit emotion recognition of static & dynamic emotional expressions (fearful, happy, neutral, disgustful, angry and sad faces)	↓ dilation for angry faces (Interpersonal facet)<br>↑ dilation for happy faces (Interpersonal and Affective facets)
Gillespie et al., 2019	N total = 73 M violent offenders; age = 38.7±11.7	TriPM total and subscales (whole sample)	Explicit recognition of emotions (morphed neutral, angry, disgustful, fearful, happy, sad, and surprised faces with different levels of intensity)	↓ pupil dilation for fearful, happy and sad faces (Meanness subscale)

Note. PCL-R = Psychopathy Checklist – Revised; PCL:SV = Psychopathy Checklist: Screening Version; PPI = Psychopathic Personality Inventory; PPI-R = Psychopathic Personality Inventory – Revised TriPM = Triarchic Psychopathy Measure; LSRP = Levenson's Self-Report Psychopathy Scale; SRP = Self-Report Psychopathy Scale; SRP-III = Self-Report Psychopathy Scale – Third Version; SRP-SF = Self-Report Psychopathy Scale – Short Form; F1 = PCL-R's Factor 1; F2 = PCL-R's Factor 2; FG = fusiform gyrus; IFG = inferior frontal gyrus; OFC = orbitofrontal cortex; PFC = prefrontal cortex; mPFC = medial PFC; dmPFC = dorsomedial PFC; vmPFC = ventromedial PFC; SMA = supplementary motor area; INS = insula; aINS = anterior INS; STS = superior temporal sulcus; pSTS = posterior STS; ACC = anterior cingulate cortex; AMY = amygdala; GM = grey matter; ERN=error-related negativity; Pe = error positivity; LPP = late-positive potential; fMRI = functional magnetic imaging; EEG = electroencephalography; ERP = event-related potentials; M age=mean age; ↓ = lower; ↑ = higher; n.s. = non-significant; M = males; F = females; HC = healthy controls; R = right; L = left.
