# Supplementary material for: Psychophysiology of facial emotion recognition in psychopathy dimensions and oxytocin’s role: A scoping review
Source: PLoS One. 2025 Jul 30;20(7):e0327764. doi: 10.1371/journal.pone.0327764 (PMC12310049; doi:10.1371/journal.pone.0327764)
Supplement: S4 Table — OT = oxytocin; in-OT = intranasal OT; IU = international units; iv-OT = intravenous OT; PL = placebo; AMY = amygdala; ACC = anterior cingulate cortex; FG = fusiform gyrus; STG = superior temporal gyrus; IFG = inferior frontal gyrus; EPN = earlier posterior negativity; INS = insula; OFC = orbitofrontal cortex; M age = mean age; ↓ = lower; ↑ = higher; ↓ a = lower activation; ↑ a higher activation; ↑ c = higher connectivity; ↓ c = lower connectivity; M = males; F = females; R = right; L = left. (PDF) [file pone.0327764.s006.pdf]

S4 Table. Studies on the effect of oxytocin intranasal administration or endogenous level on facial emotion recognition performance and functional central and peripheral nervous system correlates

Study	Sample	Intervention/Levels	Design	Task/Stimuli	Association with oxytocin
**Behavior (accuracy)**					
Campbell et al., 2014	N total = 68; 34 old M (n = 17); F (n = 17); age = 72.1±6.5); 34 young M (n = 17); F (n = 17); age = 19.7±1.8	20 IU in-OT	Double blind, PL-controlled, between-subjects	Explicit recognition of emotions (angry, disgustful, fearful, happy, sad, and neutral)	↑ accuracy for older males (vs. younger males, younger females and older females)
Di Simplicio et al., 2009	N total = 29 M; PL (n = 15) age = 23.1±2.7; in-OT (n = 14); age = 24.3±3.4	24 IU in-OT	Double blind, PL-controlled, between-subjects	Explicit recognition of emotions (morphed angry, disgustful, fearful, happy, sad and surprised faces with different intensities); Cambridge face memory test; Attentional visual probe	↑ response time for fearful faces (vs. PL) ↓ misclassification of surprised faces as a disgustful or sad expression, and neutral as a sad expression (vs. PL)
Domes et al., 2013a	N total = 62 M; age = 24.0±2.5	24 IU in-OT	Double blind, PL-controlled, between-subjects	Explicit recognition of dynamic emotions (neutral to happy and angry faces); early exploration phase and	↑ response time for happy faces (vs. PL)

Study	Sample	Intervention/Levels	Design	Task/Stimuli	Association with oxytocin
				emotion recognition phase	
Feeser et al., 2014	N total = 82 M; age = 27.9±4.7	24 IU in-OT	Double blind, PL-controlled, between-subjects	Explicit recognition of emotions (fearful, angry, disgustful, sad, happy, surprised and neutral)	↑ accuracy for fearful faces (vs. PL)
Gamer et al., 2010	N total = 46 M; age = 25±3.7	24 IU in-OT	Double blind, PL-controlled, between-subjects	Explicit recognition of emotions (fearful, happy and neutral faces, downward of upward of fixation cross, such that eyes or mouth appeared at the location of the fixation cross)	Accuracy and response time: n.s.
Kanat et al., 2014	N total = 49 M; age = 23.6±2.8	24 IU in-OT	Double blind, PL-controlled, between-subjects	Explicit recognition of emotions (angry, happy and neutral faces, followed by a masked face); short stimulus or long stimulus presentation	Accuracy and response time: n.s.

Study	Sample	Intervention/Levels	Design	Task/Stimuli	Association with oxytocin
Kis et al., 2013	N total = 56 M; age = 23.0±3.3	24 IU in-OT	Double blind (half single-blind), PL-controlled, Between-subjects	Explicit recognition of emotions (happy, neutral, angry and fearful faces); Valence rating (positive or negative)	↑ positive bias in negative emotions recognition (vs. PL)
Leknes et al., 2013	N total = 40; M (n = 20); F (n = 20); age = 26	40 IU in-OT	Double blind, PL-controlled, crossover	Explicit recognition of emotions: Implicitly “hybrid” (happy-neutral and angry-neutral faces) and explicit angry and happy faces; concomitant tactile stimulation	↑ recognition accuracy for angry and happy faces (vs. PL)
Lischke et al., 2012	N total = 47 M; age = 26.1±3.4	24 IU in-OT	Double blind, PL-controlled, between-subjects	Explicit recognition of dynamic emotions (neutral to happy, angry, sad and fearful faces)	↓ recognition threshold for angry faces (vs. PL) ↑ recognition accuracy for fearful faces (vs. PL)
Marsh et al., 2010	N total = 50 M (n = 29) and F (n = 21); age = 26.4	24 IU in-OT	Double blind, PL-controlled, between-subjects	Explicit recognition of emotions (morphed angry, disgustful, fearful, happy, sad and surprised faces with different intensities)	Response time: n.s. ↑ accuracy for happy faces (in males and females) (vs. PL)

Study	Sample	Intervention/Levels	Design	Task/Stimuli	Association with oxytocin
Matsunaga et al., 2020	N total = 51 F; primiparous mothers, breastfeeding 2- to 9-month-old infants	Salivary OT	N.A.	Explicit recognition of emotions (neutral, angry and happy faces); previous breastfeeding vs. holding the infant	↑ accuracy for happy faces (vs. angry) in breastfeeding<br>Response time: n.s.<br>↓ accuracy for angry faces (vs. neutral) in breastfeeding
Peltola et al., 2018	N total = 52 mothers; mothers (age = 31.9±5.0); infants (age = 14.5±1.2 months)	24 IU in-OT	Double blind, PL-controlled, within-subjects	Explicit recognition of emotions (happy and sad/distressed adult and infant faces)	Response time: n.s.
Perry et al., 2013	N total = 30; M (n = 19); F (n = 11); age = 38.9±10.6	24 IU in-OT	Double blind, PL-controlled, within-subjects	Explicit recognition of emotions: disgustful faces placed on body images in emotional contexts (disgustful, angry, sad and fearful)	Response time: n.s.<br>↑ accuracy of disgustful faces in the angry body context (vs. PL)
Prehn et al., 2013	N total = 47 M; age = 26.1±3.4	24 IU in-OT	Double blind, PL-controlled, between-subjects	Explicit dynamic recognition of emotions (neutral to happy, angry, sad and fearful faces)	Accuracy: n.s.<br>↓ recognition threshold for angry faces (vs. PL)

Study	Sample	Intervention/Levels	Design	Task/Stimuli	Association with oxytocin
Quintana et al., 2019b	N total = 57 M; age = 23.8±3.3	8 IU, 24 IU in-OT;1 IU iv-OT	Double blind, double-dummy, PL-controlled, crossover	Explicit recognition of emotions (happy, angry and neutral/ambiguous faces; shapes of different colors); Emotional ratings (emotion intensity)	Emotional ratings: n.s.
Quintana et al., 2016	N total = 57 M; age = 18-35	8 IU, 24 IU in-OT;1 IU iv-OT	Double blind, double-dummy, PL-controlled, crossover	Explicit recognition of emotions (happy, angry and neutral/ambiguous faces; shapes of different colors); Emotional ratings (emotion intensity)	Emotional ratings: n.s.
Schulze et al., 2011	N total = 56 M; age = 24.3±3.1	24 IU in-OT	Double blind, PL-controlled, between-subjects	Explicit recognition of emotions (angry, happy and neutral faces intercalated with a masked neutral face)	↑ accuracy for emotional regardless of valence and presentation time (vs. PL) ↑ accuracy for happy faces (vs. angry)
Spengler et al., 2017	N total = 116 M; age = 24.7±4.4	12 IU, 24 IU, 48 IU in-OT	Double blind, PL-controlled, crossover	Explicit recognition of emotions (morphed fearful, happy and	↓ Emotional bias in the recognition of neutral (vs. PL)

Study	Sample	Intervention/Levels	Design	Task/Stimuli	Association with oxytocin
				neutral faces with different intensities and initial fixation – mouth vs. eyes)	
Tollenaar et al., 2013	N total = 20 M; age = 21±3	24 IU in-OT	Double blind, PL-controlled, crossover	Implicit emotional gaze cueing task (happy, neutral and fearful faces)	↑ gaze cued orienting of attention for happy and fearful faces (vs. PL)
Van der Donck et al., 2022	N total = 31 M; age = 22.8±2.4	24 IU in-OT	Double blind, PL-controlled, crossover	Implicit emotional face-matching task (angry, happy, disgustful, sad, fearful, and surprised faces)	Accuracy: n.s.<br>Response time: n.s.
**fMRI**					
Domes et al., 2007	N total = 13 M; age = 25.7±2.9	24 IU in-OT	Double blind, PL-controlled, within-subjects	Implicit recognition of emotions (morphed happy, fearful, angry and neutral faces with different intensities)	↓ R AMY for all emotions (vs. PL)
Kanat et al., 2015	N total = 43 M; PL (n = 21); age = 23.9±2.7; in-OT (n = 22); age = 24.3±3.4	24 IU in-OT	Double blind, PL-controlled, between-subjects	Implicit emotional recognition task (masked fearful and happy eyes, followed by the target	↓ R AMY for fearful masked eyes (vs. happy)<br>↓ L ACC and L mid-temporal gyrus for fearful

Study	Sample	Intervention/Levels	Design	Task/Stimuli	Association with oxytocin
				eyes in neutral faces; scrambled eye components as control stimuli)	eyes (vs. happy) across stimuli
Kanat et al., 2014	N total = 49 M; age = 23.6±2.8	24 IU in-OT	Double blind, PL-controlled, between-subjects	Explicit recognition of emotions (angry, happy and neutral faces, followed by a masked face); short stimulus or long stimulus presentation	↓ bilateral AMY for eyes (vs. mouth) of masked angry faces, and for mouth (vs. eyes) of masked happy faces, in short stimulus<br>↓ medial superior frontal gyrus for mouth (vs. eyes) of happy faces<br>↓ inferior occipital regions, temporal part of the ventral stream and brainstem regions for eyes (vs. mouth) of masked angry faces for short stimuli<br>↓ mid-temporal gyrus, superior colliculi, and

Study	Sample	Intervention/Levels	Design	Task/Stimuli	Association with oxytocin
					striate to eyes (vs. mouth) of masked angry faces for long stimuli<br>↓ L FG to eyes (vs. mouth) of masked angry faces for all stimuli<br>↓ connectivity of L AMY with L FG to eyes of angry faces (vs. eyes of neutral)
Ma et al., 2022	N total = 65 F; nulliparous women; age = 18-26	24 IU in-OT	Double blind, PL-controlled, between-subjects	Implicit recognition of emotions (joy, neutral and crying infant faces)	↓ R AMY and bilateral insula for crying faces (vs. PL) only in women with attachment anxiety<br>↑ bilateral AMY and left inferior OFC for crying faces (vs. PL) only in women with attachment avoidance
Quintana et al., 2016	N total = 57 M; age = 18-35	8 IU, 24 IU in-OT;1 IU iv-OT	Double blind, double-dummy, PL-controlled, crossover	Explicit recognition of emotions (happy, angry and neutral/ambiguous	↓ a in R AMY for angry, happy and neutral faces as well shapes in 8 IU

Study	Sample	Intervention/Levels	Design	Task/Stimuli	Association with oxytocin
				faces; shapes of different colors); Emotional ratings (emotion intensity)	intervention condition (vs. PL)
Spengler et al., 2017	N total = 116 M; age = 24.7±4.4	12 IU, 24 IU, 48 IU in-OT	Double blind, PL-controlled, crossover	Explicit recognition of emotions (morphed fearful, happy and neutral faces with different intensities and initial fixation – mouth vs. eyes)	↓ a in L AMY for fearful faces, moderated by fear intensity (in 24 IU) (vs. PL)
**EEG**					
Peltola et al., 2018	N total = 52 mothers; mothers (age = 31.9±5.0); infants (age = 14.5±1.2 months)	24 IU in-OT	Double blind, PL-controlled, within-subjects	Explicit recognition of emotions (happy and sad/distressed adult and infant faces)	↑ N170 amplitude for infant (vs. adult) and sad faces (vs. happy)<br>↑ LPP amplitude for infant faces (vs. adult)
Tillman et al., 2019	N total = 21 M; age = 25.2±3.7	60 IU in-OT	Double blind, PL-controlled, within-subjects	Experiment 1: Implicit dynamic faces recognition task (continuum neutral-fear or fear-neutral faces);	↑ N170 amplitude and ↓ latency for fearful faces (vs. neutral) (Experiment 1) ↓ latency for eyes (vs.

Study	Sample	Intervention/Levels	Design	Task/Stimuli	Association with oxytocin
				Experiment 2: Attention task (neutral faces and houses)	mouth) overall (Experiment 2) Non-significant effects on P100 and EPN amplitude or latency for each condition (Experiment 1 and 2)
Van der Donck et al., 2022	N total = 31 M; age = 22.8±2.4	24 IU in-OT	Double blind, PL-controlled, crossover	Implicit recognition of emotions (angry, fear and happy faces)	Neural sensitivity in occipito-temporal and medial-occipital regions through frequency-tagging EEG: n.s.
**Eye-gazing**					
Domes et al., 2013a	N total = 62 M; age = 24.0±2.5	24 IU in-OT	Double blind, PL-controlled, between-subjects	Explicit recognition of dynamic emotions (neutral to happy and angry faces); early exploration phase and emotion recognition phase	↑ eye-gazing for neutral (early exploration phase) and ↑ eye-gazing overall (emotion recognition phase) (vs. PL)

Study	Sample	Intervention/Levels	Design	Task/Stimuli	Association with oxytocin
Domes et al., 2013b	N total = 69 M; age = 24.0±3.1	24 IU in-OT	Double blind, PL-controlled, between-subjects	Implicit recognition of emotions: Dot-probe paradigm (angry, happy and neutral faces); probe localization in congruent (same as the emotional face) or incongruent (same as the neutral) contexts; short and long duration of presentation	↑ attention shift to happy faces (short duration) (vs. PL)
Lischke et al., 2012	N total = 47 M; age = 26.1±3.4	24 IU in-OT	Double blind, PL-controlled, between-subjects	Explicit recognition of dynamic emotions (neutral to happy, angry, sad and fearful faces)	↑ eye-gazing for sad faces, recognized at lower intensity (vs. PL)
**Pupillometry**					
Burley & Daughters, 2020	N total = 94 M; age = 19.7±1.7	24 IU in-OT	Double blind, PL-controlled, mixed-design	Explicit dynamic recognition of emotions (neutral to happy, sad, fearful and angry faces)	n.s.
Leknes et al., 2013	N total = 40; M (n = 20); F (n = 20); age = 26	40 IU in-OT	Double blind, PL-controlled, crossover	Explicit recognition of emotions: Implicitly “hybrid” (happy-neutral	↑ pupil dilation for participants with low sensitivity (vs. high)

Study	Sample	Intervention/Levels	Design	Task/Stimuli	Association with oxytocin
				and angry-neutral faces) and explicit angry and happy faces; concomitant tactile stimulation	towards differences between the implicit angry and happy faces ↑ stimulus-induced pupil dilation overall (vs. PL)
Prehn et al., 2013	N total = 47 M; age = 26.1±3.4	24 IU in-OT	Double blind, PL-controlled, between-subjects	Explicit dynamic recognition of emotions (neutral to happy, angry, sad and fearful faces)	↑ pupil dilation to happy faces and male faces (vs. female) (vs. PL)
**fMRI with eye-gazing or pupillometry**					
Domes et al., 2010	N total = 16 F; age = 24.2±2.5	24 IU in-OT	Double blind, PL-controlled, within-subjects, crossover	Implicit recognition of emotions (angry, fearful, happy and neutral faces)	Fixation pattern: n.s. ↑ bilateral FG, L STG and L AMY for fearful faces (vs. neutral) ↑ bilateral IFG for angry faces (vs. neutral) ↑ L FG and R IFG for happy faces (vs. neutral)
Gamer et al., 2010	N total = 46 M; age = 25±3.7	24 IU in-OT	Double blind, PL-controlled, between-subjects	Explicit recognition of emotions (fearful, happy and neutral faces, downward of upward of	↓ L AMY for fearful faces and ↑ L AMY for happy faces (vs. PL)

Study	Sample	Intervention/Levels	Design	Task/Stimuli	Association with oxytocin
				fixation cross, such that eyes or mouth appeared at the location of the fixation cross)	↑ eye-gazing irrespective of emotion (vs. PL)<br>Positive correlation between eye-gazing and R posterior AMY irrespective of emotion (vs. PL)<br>↑ connectivity between AMY and superior colliculus when gaze-related (vs. PL)
Quintana et al., 2019b	N total = 57 M; age = 23.8±3.3	8 IU, 24 IU in-OT;1 IU iv-OT	Double blind, double-dummy, PL-controlled, crossover	Explicit recognition of emotions (happy, angry and neutral/ambiguous faces; shapes of different colors);<br>Emotional ratings (emotion intensity)	↓ pupil dilation overall (in 8 IU) (vs. PL)<br>Positive correlation between R AMY and pupil dilation for angry, neutral, happy faces and shapes (in 8 IU) (vs. PL)

Note. OT = oxytocin; in-OT = intranasal OT; IU = international units; iv-OT = intravenous OT; PL = placebo; AMY = amygdala; ACC = anterior cingulate cortex; FG = fusiform gyrus; STG = superior temporal gyrus; IFG = inferior frontal gyrus; EPN = earlier posterior negativity; INS = insula; OFC = orbitofrontal cortex; M age = mean age; ↓ = lower; ↑ = higher; n.s. = non-significant; M = males; F = females; R = right; L = left.
