# Supplementary material for: Psychophysiology of facial emotion recognition in psychopathy dimensions and oxytocin’s role: A scoping review
Source: PLoS One. 2025 Jul 30;20(7):e0327764. doi: 10.1371/journal.pone.0327764 (PMC12310049; doi:10.1371/journal.pone.0327764)
Supplement: S5 Table — Q1. Were the hypothesis and objectives of the study clearly described? Q2. Have the authors used methods that can test their hypothesis? Q3. Were the recruitment procedure and eligibility criteria for the participants clearly described (e.g., ethnicity, age range, gender, neuropsychiatric and other medical conditions, handedness)? Q4. Was there an effort to account for demographic or neuropsychological variability amongst participants (e.g., age, gender, handedness, IQ, level of education, smoking, alcohol/drug use and, for cases, duration of illness and medication status)? Q5. Did the study include an a priori power calculation to define sample sizes? Q6. Was a valid cognitive task assigned to elicit the desired effects in the participants and it was clearly described? Q7. Was the assessment of psychopathy performed with valid measurement tools and clearly described, when applied? Q8. Were aspects related to oxytocin administration or measurement adequate and clearly described, when performed (e.g., dose, administration method, time of administration, laboratory technique for oxytocin quantification)? Q9. In studies with central and/or peripheral nervous system activity assessment, the technique, respective parameters and preprocessing were outlined? Q10. Was the statistical methodology clearly explained? Q11. Was a consensual statistical significance threshold set (i.e.,., p < 0.05 after correction for multiple comparisons)? Q12. Were effect sizes reported for significant results (e.g., Cohen’s d, Pearson’s correlation, odds ratio or risk ratios)? The general quality of the study was obtained by dividing the sum of the 12 items’ scores by the maximum sum applicable to each study. N/A = Not applicable. (PDF) [file pone.0327764.s007.pdf]

S5 Table. Quality scores for each study included in this review, assessed from 0 to 3 across the preestablished 12-criteria list

Study	Q1	Q2	Q3	Q4	Q5	Q6	Q7	Q8	Q9	Q10	Q11	Q12	General quality score
**Studies on the association of psychopathy and facial emotion recognition performance and functional central and peripheral nervous system correlates**													
Almeida et al., 2014	2	3	2	2	0	3	3	N/A	3	2	2	3	75.8%
Beussink et al., 2020	1	3	2	1	0	2	3	N/A	N/A	1	3	3	63.3%
Brislin & Patrick, 2019	3	3	1	1	0	2	3	N/A	3	3	3	3	75.8%
Burley et al., 2017	3	3	1	1	3	3	3	N/A	3	3	3	3	87.9%
Burley et al., 2019	2	3	3	3	3	3	3	N/A	3	3	1	3	90.9%
Carré et al., 2013	3	3	2	1	0	3	3	N/A	3	3	3	3	81.8%
Contreras-rodríguez et al., 2014	2	3	2	3	0	3	3	N/A	3	3	2	3	81.8%
Dargis et al., 2018	2	3	3	3	0	3	3	N/A	3	3	2	3	84.9%
Decety et al., 2014	2	3	2	3	0	3	2	N/A	2	2	3	2	72.7%
Deeley et al., 2006	3	3	3	2	0	3	1	N/A	2	3	2	3	75.8%
Dolan & Fullam, 2006	3	3	3	3	0	2	3	N/A	N/A	2	2	3	80%
Dolan & Fullam, 2004	3	3	3	3	0	3	3	N/A	N/A	2	2	3	83.3%
Eisenbarth et al., 2013	3	3	2	3	0	3	3	N/A	3	3	3	3	87.9%
Eisenbarth et al., 2008	1	3	3	3	0	3	3	N/A	N/A	2	3	3	80%
Faith et al., 2023	3	3	3	2	0	2	3	N/A	N/A	2	3	3	80%

Study	Q1	Q2	Q3	Q4	Q5	Q6	Q7	Q8	Q9	Q10	Q11	Q12	General quality score
Faith et al., 2022	2	3	1	2	1	2	2	N/A	N/A	2	2	3	66.7%
Gehrer et al., 2019	3	3	2	1	3	3	3	N/A	3	3	2	3	87.9%
Gillespie et al., 2019	2	3	2	3	0	3	3	N/A	3	3	3	3	84.9%
Gillespie et al., 2017	3	3	2	1	0	3	3	N/A	3	3	3	3	81.8%
Gillespie et al., 2015	2	3	1	1	0	2	3	N/A	3	3	3	3	72.7%
Glass & Newman, 2006	3	2	2	1	0	2	3	N/A	N/A	2	1	3	63.3%
Gordon et al., 2004	2	3	1	1	0	3	1	N/A	2	3	3	3	66.7%
Hansen et al., 2008	2	3	2	1	0	2	2	N/A	N/A	2	1	3	60%
Igoumenou et al., 2017	2	3	3	3	0	3	3	N/A	N/A	1	1	3	73.3%
Jusyte & Schönenberg, 2017	2	2	2	1	0	3	3	N/A	N/A	2	2	3	66.7%
Khvatskaya & Lenzenweger, 2016	3	3	3	3	0	3	3	N/A	N/A	3	1	3	83.3%
Kosson et al., 2002	3	2	3	3	0	2	1	N/A	N/A	2	2	3	70%
Kranefeld & Blickle, 2022	2	2	1	1	0	2	2	N/A	N/A	1	2	2	50%
Kuin et al., 2017	2	3	3	2	3	3	3	N/A	N/A	3	3	3	93.3%
Künecke et al., 2018	3	3	3	1	0	3	3	N/A	N/A	3	3	3	83.3%
Mier et al., 2014	2	3	3	3	0	3	1	N/A	3	3	2	3	78.8%
Mowle et al., 2019	2	2	2	2	0	2	3	N/A	3	2	1	3	66.7%
Munro et al., 2007	1	3	2	1	0	3	3	N/A	3	2	2	3	69.7%
Olderbak et al., 2018	1	3	2	1	0	3	3	N/A	N/A	2	3	3	70%

Study	Q1	Q2	Q3	Q4	Q5	Q6	Q7	Q8	Q9	Q10	Q11	Q12	General quality score
Oliver et al., 2015	3	3	2	2	0	3	3	N/A	N/A	3	1	3	76.7%
Pera-Guardiola et al., 2016	2	3	2	3	0	2	3	N/A	3	3	3	3	81.8%
Pham & Philippot, 2010	1	2	2	2	0	2	3	N/A	N/A	3	3	3	70%
Philipp-Wiegmann et al., 2017	3	3	3	2	0	2	2	N/A	N/A	1	2	3	70%
Schönenberg & Jusyte, 2014	3	3	2	1	0	3	3	N/A	N/A	1	1	3	66.7%
**Studies on the effect of oxytocin intranasal administration or endogenous level on facial emotion recognition performance and functional central and peripheral nervous system correlates**													
Burley & Daughters, 2020	2	3	2	1	0	3	N/A	3	3	2	3	3	75.8%
Campbell et al., 2014	2	3	2	1	0	2	N/A	3	N/A	1	1	3	60%
Di Simplicio et al., 2009	2	3	3	1	0	3	N/A	3	N/A	2	3	3	76.7%
Domes et al., 2013a	2	3	2	2	0	2	N/A	3	2	2	2	3	69.7%
Domes et al., 2013b	3	3	1	2	0	3	N/A	3	2	2	1	3	69.7%
Domes et al., 2010	2	3	1	1	0	3	N/A	3	3	3	3	3	75.8%
Domes et al., 2007	2	3	1	1	0	3	N/A	3	2	1	3	3	66.7%
Feeser et al., 2014	3	3	2	1	0	3	N/A	3	N/A	3	3	3	80%
Gamer et al., 2010	2	3	2	1	0	3	N/A	3	2	3	3	3	75.8%
Kanat et al., 2015	3	3	3	1	0	2	N/A	3	3	2	3	3	78.8%
Kanat et al., 2014	2	3	2	1	0	3	N/A	3	3	2	3	3	75.8%
Kis et al., 2013	2	2	3	1	0	2	N/A	3	N/A	3	2	3	70%

Study	Q1	Q2	Q3	Q4	Q5	Q6	Q7	Q8	Q9	Q10	Q11	Q12	General quality score
Leknes et al., 2013	2	2	2	1	0	2	N/A	3	3	2	1	3	63.6%
Lischke et al., 2012	2	2	2	1	0	2	N/A	3	3	2	1	3	63.6%
Ma et al., 2022	3	3	2	1	0	3	N/A	3	3	3	3	3	81.8%
Marsh et al., 2010	3	3	3	3	0	3	N/A	3	N/A	3	2	3	86.7%
Matsunaga et al., 2020	1	3	2	1	0	3	N/A	3	N/A	3	2	3	70%
Peltola et al., 2018	2	3	3	1	0	3	N/A	3	3	2	1	3	72.7%
Perry et al., 2013	2	2	1	1	0	2	N/A	3	N/A	2	2	3	60%
Prehn et al., 2013	2	3	2	2	0	3	N/A	3	3	3	1	3	75.8%
Quintana et al., 2019b	1	3	2	1	0	2	N/A	2	2	3	2	3	63.6%
Quintana et al., 2016	3	3	2	2	0	3	N/A	3	3	3	3	3	84.9%
Schulze et al., 2011	2	2	2	1	3	2	N/A	3	N/A	2	3	3	76.7%
Spengler et al., 2017	3	3	1	1	0	3	N/A	3	3	2	3	3	75.8%
Tillman et al., 2019	3	3	3	1	0	3	N/A	3	3	3	3	3	84.9%
Tollenaar et al., 2013	3	3	2	1	0	3	N/A	3	N/A	3	2	3	76.7%
Van der Donck et al., 2022	3	3	2	1	0	3	N/A	3	3	2	2	3	75.8%

Note. **Q1.** Were the hypothesis and objectives of the study clearly described? **Q2.** Have the authors used methods that can test their hypothesis? **Q3.** Were the recruitment procedure and eligibility criteria for the participants clearly described (e.g., ethnicity, age range, gender, neuropsychiatric and other medical conditions, handedness)? **Q4.** Was there an effort to account for demographic or neuropsychological variability amongst participants (e.g., age, gender, handedness, IQ, level of education, smoking, alcohol/drug use and, for cases, duration of illness and medication status)? **Q5.** Did

the study include an a priori power calculation to define sample sizes? **Q6.** Was a valid cognitive task assigned to elicit the desired effects in the participants and it was clearly described? **Q7.** Was the assessment of psychopathy performed with valid measurement tools and clearly described, when applied? **Q8.** Were aspects related to oxytocin administration or measurement adequate and clearly described, when performed (e.g., dose, administration method, time of administration, laboratory technique for oxytocin quantification)? **Q9.** In studies with central and/or peripheral nervous system activity assessment, the technique, respective parameters and preprocessing were outlined? **Q10.** Was the statistical methodology clearly explained? **Q11.** Was a consensual statistical significance threshold set (i.e.., $p < 0.05$ after correction for multiple comparisons)? **Q12.** Were effect sizes reported for significant results (e.g., Cohen's d, Pearson's correlation, odds ratio or risk ratios)?

The general quality of the study was obtained by dividing the sum of the 12 items' scores by the maximum sum applicable to each study. N/A = Not applicable
